# (En)Abling Architectural Research: Co‐Designing With People With Intellectual Disabilities

**DOI:** 10.1111/jar.70275

**Published:** 2026-07-01

**Authors:** Menatalla Kasem, Zarah Kaleem, Sam Clark, Dikaios Sakellariou

**Affiliations:** ^1^ Architecture Department, Faculty of Engineering Zagazig University Zagazig Egypt; ^2^ Cardiff People First Cardiff Wales; ^3^ The Welsh School of Architecture Cardiff University Cardiff Wales; ^4^ Department of Health Sciences European University Nicosia Cyprus

**Keywords:** architecture, co‐design, inclusive design, intellectual disability, participatory research

## Abstract

**Background:**

People with intellectual disabilities remain under‐represented in architectural design and research. This paper draws on a doctoral study that explored how people with intellectual disabilities experience shopping centres. It aims to present an inclusive methodological framework that supports their meaningful participation in architectural research.

**Methods:**

The study followed a Participatory Action Research approach supported by ethnographic methods. Data was generated through interviews, walking interviews, focus groups and co‐design workshops and the data was analysed using reflexive thematic analysis.

**Results:**

The analysis identified enabling measures that shaped an inclusive research environment, including collaboration with a self‐advocacy organisation, the involvement of a co‐researcher with lived experience, the use of prompts and flexible timelines.

**Conclusion:**

The resulting framework offers practical guidance for researchers seeking to embed inclusive approaches within architectural research and related fields. The article is co‐authored by academic researchers and a co‐researcher with lived experience of an intellectual disability.

## Abstract (plain language summary)

**Lay Summary**

- This article explains how architectural research can be made more inclusive by working with people with intellectual disabilities as partners, rather than doing research for them.
- A self‐advocacy organisation helped with the research, and a co‐researcher with an intellectual disability was employed.
- Together, we created a new way of doing research that supports people with intellectual disabilities to take part.
- This new way can help other researchers include people with intellectual disabilities in architectural research.

## 1 Introduction

The built environment plays a crucial role in enabling people to participate in society. For disabled people, architectural design can either facilitate inclusion or reinforce exclusion. The United Nations Convention on the Rights of Persons with Disabilities (CRPD) affirms that all individuals have the right to equal access to the physical environment, transportation and public facilities (United Nations 2006a), reinforcing that accessibility is not a privilege but a human right (United Nations 2006b). Disability, as defined by the World Health Organization (2022), arises from the interaction between an individual's health condition and their physical and social environment. Globally, over 1 billion people live with some form of disability, with around 110–190 million adults facing significant functional difficulties (World Health Organization 2022). In the United Kingdom, approximately 1.5 million people have an intellectual disability (Mencap 2022).

A person with an intellectual disability might have some difficulty understanding complicated information, learning some skills or looking after themselves (The National Health Service 2022). As a result, they may face barriers to accessing, using and enjoying public spaces (Castell 2008). Designing environments that support independence and reduce marginalisation is, therefore, critical (Salmi 2007; Kasem et al. 2023). Thoughtfully designed spaces can reduce stress, promote autonomy and make the everyday experiences of people with intellectual disabilities more positive and empowering (González et al. 2020; Park et al. 2020).

However, designers rarely reflect the diversity of the people who use buildings. According to the Architects Registration Board (ARB) report on the architects' profession in 2022, only 1% of architects in the United Kingdom are disabled (Architects Registration Board 2023). As a result, architects may be unaware of the varied accessibility needs of different users, including people with intellectual disabilities. This gap highlights the importance of working collaboratively with disabled people to ensure that design and research processes are informed by lived experience (Castell 2012; Gaudion et al. 2015; Hendriks et al. 2015; Sarmiento‐Pelayo 2015).

Yet, people with intellectual disabilities are still too often positioned as objects of research rather than as experts with lived experience. They are usually overlooked in architectural design and research (Castell 2008; Mathers 2008; World Health Organization 2011; Park et al. 2020). Even when efforts are made to include disabled voices, participation is frequently limited or tokenistic, rather than a source of insight (House of Commons Women and Equalities Committee 2017).

This article draws on a doctoral study conducted in Cardiff, Wales, which explored how people with intellectual disabilities experience architectural barriers in shopping centres. The study combined a Participatory Action Research (PAR) approach with ethnography, working in partnership with a self‐advocacy organisation and employing a co‐researcher with an intellectual disability. Through iterative engagement across interviews, walking interviews, focus groups and co‐design workshops, the study developed a methodological framework to support inclusive architectural research. This article focuses specifically on this framework, outlining the enabling measures that supported meaningful participation and discussing their implications for inclusive research practice.

## 2 Research Problem

The spatial and experiential needs of people with intellectual disabilities remain insufficiently understood, often resulting in environments that are retrofitted reactively rather than designed inclusively from the outset (Castell 2008, 2012; Finlayson et al. 2015; Hafez et al. 2021), although physical accessibility has gained prominence in architectural discourse since the 1970s (Hamraie 2017). This gap reflects deeper systemic issues at the intersection of societal attitudes, regulatory frameworks and architectural practice.

Firstly, societal stigma casts people with intellectual disabilities as passive recipients rather than active contributors to knowledge production (Castell 2014; Jarrett and Tilley 2022). Traditional research practices can reinforce epistemic injustice by discounting the perspectives and interpretations of disabled people (Felder 2025), highlighting the need for genuinely inclusive and co‐produced approaches. Secondly, the general and minimal nature of building codes/regulations often fails to represent the needs of people with intellectual disabilities adequately, leading to ableist assumptions embedded within architectural education and regulations (Imrie and Hall 2001; Salmi 2007; Castell 2008; Boys 2014; Evans 2015). Finally, there is a disconnect between architectural professionals and the diverse communities they serve; a survey revealed that only 8% of architects consistently consider the needs of people with intellectual disabilities in their designs (Imrie and Hall 2001).

This article responds to these challenges by presenting a participatory methodological framework designed to support more inclusive architectural research. The framework aims to address the epistemic exclusion of people with intellectual disabilities by creating conditions for meaningful involvement throughout the research process. It offers researchers concrete ways to make architectural research fairer, more inclusive and more closely aligned with the lived experiences and priorities of people with intellectual disabilities.

## 3 Collaborative Research Approaches

Collaborative research involves working with participants rather than studying them and includes approaches such as participatory, emancipatory and inclusive research (Peuravaara 2015). Participatory research emphasises collaboration between researchers and participants, actively involving them in the research process (Cornwall and Jewkes 1995), while emancipatory research aims to promote social change by empowering participants to challenge societal inequalities and take control of the research process (Gilbert 2004). On the other hand, inclusive research involves deeper participation of people with intellectual disabilities beyond mere involvement (Walmsley and Johnson 2003).

PAR combines action and reflection, enabling people who experience an issue to investigate it and contribute to social change (Cornish et al. 2023). It challenges traditional power dynamics by positioning participants as co‐researchers who help generate knowledge (Cornwall and Jewkes 1995; Gilbert 2004). One of the most important features of PAR, when engaging with marginalised or vulnerable individuals, is its hands‐on nature and its capacity to empower people to produce information and share knowledge on their own terms, using familiar symbols, language or art forms (Kindon et al. 2007), making it ideal for doing research with people with intellectual disabilities.

The active involvement and engagement of research participants define the inclusive and collaborative research practice that sets PAR apart from conventional research, where the outside researcher sets the agenda, decides on the questions to be asked and implements the survey for later analysis (Kindon et al. 2007; Schubotz 2019). The methodological uniqueness of PAR is in its nature of building relationships between people to connect theory and practice (Schubotz 2019).

### 3.1 Co‐Design With People With Intellectual Disabilities

Collaborative approaches are increasingly recognised as essential within the built‐environment professions. Guidance such as the Royal Institute of British Architects (RIBA) ‘Inclusive Design Overlay’ emphasises that inclusion must be embedded across all project stages and treated as a shared responsibility (Royal Institute of British Architects 2023). Meaningful dialogue between designers and users is central to this shift, requiring researchers to observe and understand how people interact with spaces in real‐world contexts.

Engaging disabled people in co‐design aligns with the United Nations CRPD call to view disabled people as subjects with rights who actively participate in decisions affecting their lives (United Nations 2006c), which reflects the social model of disability. Including them early in the design process makes their needs, desires and priorities visible and actionable, thereby improving the relevance of design outcomes (González et al. 2020).

Co‐design positions people with intellectual disabilities as experts by experience whose insights are crucial for achieving accessibility and inclusivity (González et al. 2020). It is an approach to designing with people rather than for them. It is particularly effective when individuals with lived experience, communities and professionals work together to address shared concerns (McKercher 2020). Co‐design is inherently flexible; it draws on creative and participatory methods that allow people to contribute using their own knowledge, skills and preferred modes of expression (McKercher 2020; Zamenopoulos and Alexiou 2018). Rather than following a fixed set of steps, co‐design relies on adaptable principles that can be tailored to different groups and contexts.

Research shows that co‐design with people with intellectual disabilities enriches both the design process and the knowledge it produces (Sarmiento‐Pelayo 2015; Sitbon and Farhin 2017; González et al. 2020). However, enacting co‐design requires careful attention to accessibility, facilitation and power‐sharing. Meaningful participation does not happen automatically; it depends on how activities are designed, supported and adapted in response to participants' needs.

While there are important examples of inclusive and participatory work involving people with intellectual disabilities in spatial, landscape and urban design, for example (Mathers 2008; Mathers et al. 2011; Thwaites et al. 2023), such approaches remain uncommon within architectural research. Existing studies have demonstrated the value of co‐production and empowerment processes, yet there is limited methodological guidance on how to adapt architectural research tools, environments and facilitation strategies to support meaningful participation by people with intellectual disabilities. The following section, therefore, explores the key challenges and opportunities involved in co‐designing with people with intellectual disabilities, drawing on existing research from different disciplines.

#### 3.1.1 Challenges and Opportunities of Co‐Designing With People With Intellectual Disabilities

A intellectual disability does not impede an individual's ability to have aspirations, emotions or ambitions (Mencap 2019). Many people with intellectual disabilities are capable of working, living independently, forming relationships and achieving academic qualifications (The National Health Service 2022). Their engagement in research is equally feasible but requires thoughtful support to navigate challenges (Walmsley 2004). Hence, traditional research methods may not be suitable; they require adaptation to meet the specific communication and cognitive needs of participants (Gaudion et al. 2014, 2015; Hendriks et al. 2015). For example, some may experience difficulties with verbal expression, which can impact their ability to convey needs and ideas in words.

Consequently, engaging with people with intellectual disabilities often requires a slower, more deliberate approach, which can conflict with the usual time constraints in typical research projects (Fudge Schormans et al. 2019; Thomas 2024). Limited‐time projects make it less likely for people with intellectual disabilities to engage fully in the research process, whilst longer timelines create an opportunity to explore new ideas and develop new skills (Fudge Schormans et al. 2019; Fraser‐Barbour et al. 2023). Hence, effective co‐design with people with intellectual disabilities requires flexible timelines that allow for continuous adaptation based on participant feedback (Gaudion et al. 2015). At the same time, involving people with intellectual disabilities in the early planning and co‐design of research processes can help shape more accessible, realistic and contextually appropriate projects, reinforcing the importance of designing with rather than for participants (Hughes and Schwartz 2024).

People with intellectual disabilities expressed various motivations for engaging in research, including enjoyment, learning opportunities, increased confidence and social interactions (Frankena et al. 2018). Furthermore, collaborative projects offer a valuable space for personal growth, skill development, making new friends and building informal networks (Fudge Schormans et al. 2019; Ćwirynkało et al. 2024).

Hendriks et al. (2015) noted that a one‐size‐fits‐all co‐design approach is rarely appropriate for people with cognitive or sensory impairments. In contrast, other research has successfully used more structured, pre‐selected methods to support participation (Barr et al. 2003; Fudge Schormans et al. 2019). Together, these perspectives show that effective co‐design with people with intellectual disabilities does not depend on a single methodological style, but on the thoughtful alignment of methods with participants' needs, preferences and communication styles. These considerations provided the foundation for the methodological approach used in this research, guiding decisions about recruitment, facilitation and the design of each data collection stage, as detailed in the next sections.

## 4 Methodology

This article draws on a doctoral study that examined how people with intellectual disabilities experience architectural barriers in shopping centres in Wales. The study adopted a PAR approach, supported by ethnographic methods, to enable sustained, collaborative engagement with participants. PAR was selected for its emphasis on empowerment, shared decision‐making and iterative development, while ethnographic techniques provided opportunities for in‐depth, contextual understanding of participants' everyday experiences.

A central feature of the methodology was the first author's dual positionality. As a non‐disabled researcher, they entered the field as an outsider, requiring deliberate efforts to build trust, establish rapport and ensure that research activities were inclusive. At the same time, their employment within a local self‐advocacy organisation created an insider position that offered sustained access to the community and opportunities for informal engagement. This dual positionality shaped the research process in significant ways, influencing how participants were approached, how activities were facilitated and how interpretations were formed.

Hence, the data was analysed using Reflexive Thematic Analysis (RTA), which acknowledges the active role of the researcher in interpreting meaning and constructing themes. This approach was well‐suited to the study's emphasis on reflexivity, positionality and participant‐centred knowledge. Analysis drew on participant insights, observational notes and the researcher's reflective field journals, allowing the dual insider–outsider perspective to inform the development of the methodological framework presented in this article.

### 4.1 Approaching the Community

Before data collection began, a collaboration was established with a self‐advocacy organisation run by and for people with intellectual disabilities in Cardiff. This partnership was essential for ensuring that the research was grounded in the priorities of the community and conducted in a way that felt safe, respectful and inclusive to participants. The organisation guided appropriate communication, cultural expectations and ethical ways of approaching potential participants, helping to shape the early direction of the study.

As part of this collaboration, the organisation recommended recruiting a co‐researcher with an intellectual disability to support the design and delivery of the project. This step was taken to strengthen the inclusivity of the research environment and to reduce power imbalances between the academic researcher and participants. Eight candidates were interviewed, and the second author was appointed based on their interest, communication strengths and familiarity with the community across Wales. Their involvement played a central role in ensuring that research activities were accessible, relevant and responsive to the needs of the community.

These steps laid the foundation for the inclusive methodological framework presented in this article. It also shaped the subsequent stages of data collection by establishing trust, shared ownership and a collaborative ethos from the outset.

## 5 Data Collection

**FIGURE 1:**
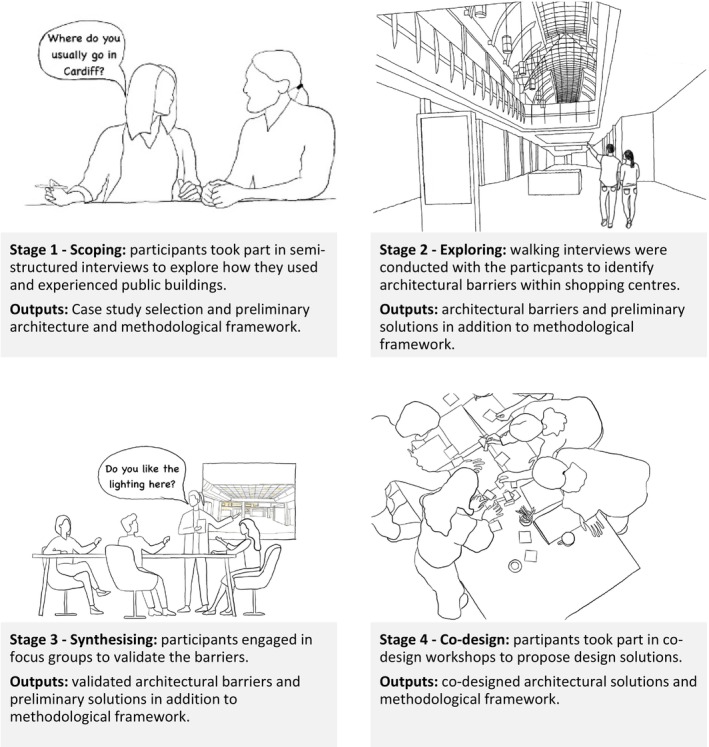
Storyboard visually illustrating the data collection stages, aims and outputs.

The research followed a qualitative approach, and the data collection was carried out in four stages, each designed to engage people with intellectual disabilities as active contributors (see Figure 1). The co‐design workshops were intentionally placed after the interviews, walking interviews and focus groups. This sequencing reflects the need first to build a shared understanding of the architectural barriers experienced by the participants in real environments. The earlier stages generated concrete, participant‐led insights about sensory, spatial and navigational challenges, which then informed the agenda and structure of the co‐design workshops.

The Research Ethics Committee at the Welsh School of Architecture, Cardiff University, granted ethical approval for the study. Recruitment for all stages was facilitated through the organisation and supported by the co‐researcher. Recruitment criteria included being 18 years or over, having an intellectual disability and living in or regularly visiting Cardiff.

### 5.1 Stage 1: Scoping

Gathered initial insights into how participants used and experienced public buildings in Cardiff. Semi‐structured interviews were conducted to explore their preferences and identify potential case study sites. These conversations informed the selection of the shopping centre used in subsequent stages. Further details, including the full interview schedule and prompts, are provided in the Supporting Information (see Figure S1).

*Sample demographics*: The sample comprised 10 participants, aged between 30 and 64 years; of these participants, eight identified as female and two as male. Several participants reported having additional conditions alongside their intellectual disability, including ADHD, autism, dyspraxia and dyslexia.

### 5.2 Stage 2: Exploring

Focused on understanding how architectural elements influenced participants' ability to access, navigate and use the selected shopping centre. Walking interviews were conducted, allowing the participants to identify barriers in real time and describe how these affected their experiences. The walking‐interview guide and cue cards are available in the Supporting Information (see Figures S2–S6 and Table S1).

*Sample demographics*: The sample included 18 participants aged 36–65 years. Eleven participants were identified as female and seven as male. Some participants reported having additional conditions alongside their intellectual disability, including ADHD, autism, borderline personality disorder, hearing impairment, dyspraxia, dyslexia and epilepsy.

### 5.3 Stage 3: Synthesising

Three focus groups were held (3–6 per group) to review and refine the barriers identified earlier. This collective reflection enabled participants to validate findings, prioritise the most significant issues and deepen the shared understanding of how architectural features shaped their experiences. Focus‐group agendas and facilitation materials are included in the Supporting Information (see Figures S9–S19).

*Sample demographics*: The sample consisted of 13 participants aged between 26 and 65 years old. Six participants identified themselves as female, and seven identified themselves as male. Some participants reported having additional conditions alongside their intellectual disability, including autism and borderline personality disorder.

### 5.4 Stage 4: Co‐Design

Involved three co‐design workshops (4–5 per workshop). Using creative and participatory methods, participants worked collaboratively to propose architectural solutions to the prioritised barriers. These workshops supported hands‐on engagement and enabled participants to express ideas using a range of accessible tools and materials. Workshop layouts, activity sheets and accessibility tools are provided in the Supporting Information (see Figures S7 and S8 and Table S2).

*Sample demographics*: The sample consisted of 14 participants aged between 26 and 65 years old. Seven identified themselves as females and seven as males.

The interview stage lasted 4 weeks, followed by an extended period of walking interviews conducted over 7 months to accommodate participant availability, energy levels and support needs. The focus groups were delivered across three sessions within 1 week, and the co‐design workshops were held across three sessions over 4 days. Overall, the fieldwork spanned approximately 18 months from the first interview to the final workshop, reflecting the flexible and iterative nature of inclusive research with people with intellectual disabilities.

Although the co‐design workshops formed the final stage of the project, co‐design principles underpinned the entire research journey. The involvement of the co‐researcher, together with ongoing collaboration with the self‐advocacy organisation, shaped decisions about recruitment, accessibility measures, interview formats, walking‐interview routes, cue‐card development and the structure of the focus groups.

Together, the four stages provided an iterative process through which people with intellectual disabilities contributed to identifying barriers, interpreting findings and generating design solutions. The structure of the data collection process also informed the development of the inclusive methodological framework presented in the following sections. This iterative, relational approach meant that each stage was adapted in response to participant feedback, resulting in methods that were more accessible, engaging and meaningful.

### 5.5 Participants Involvement Across Stages

The total number of participants across all stages was 55, of which 26 were unique participants, accounting for overlap across stages. The overlap was not intended or avoided; some participated in one stage, while others participated in all stages (see Table 1).

**TABLE 1:** Participant involvement across the four research stages arranged in alphabetical order.

Participant (pseudonym)	Stage 1: Interviews	Stage 2: Walking interviews	Stage 3: Focus groups	Stage 4: Co‐design workshops
Abbey		√		
Amelia		√		√
Camila	√			
Daisy	√	√	√	√
Daniel		√	√	√
Freya			√	
Gabriel			√	√
Gavin	√	√		
Grace	√	√	√	
Isla		√		
Jessica		√		
Julia				√
Leo			√	√
Lucy		√		
Mia	√			
Micheal	√	√	√	√
Olivia	√	√	√	
Patrick		√		√
Paul		√	√	
Richard		√		
Rose	√	√		√
Sam		√	√	√
Sebastian			√	√
Skylar			√	√
Sophia	√	√	√	√
Summer	√	√		√

## 6 Analysis

In RTA, the researcher acknowledges and reflects on the ways that their values, experiences, interests and social location inform the analysis being undertaken (Terry and Hayfield 2020). RTA depends on the researcher's subjective, active engagement with their data in relation to the research question. In this approach, themes are not perceived to ‘emerge’ from the chaos of data but are constructed, tested and refined through a series of iterative phases (Terry and Hayfield 2020).

This approach integrated participant insights with observational data, ensuring robust, participant‐centred findings. The method's iterative and reflexive nature guided the analysis, informed by both the data and the researcher's reflections. RTA was applied across all research stages, remaining sensitive to participants' voices while acknowledging the researcher's influence on the process due to their employment experience.

The reflexive approach involved six recursive steps are as follows: (i) familiarisation, (ii) generating codes, (iii) constructing candidate themes, (iv) reviewing themes, (v) naming and defining themes and (vi) writing the report (as per Terry and Hayfield 2020; Braun and Clarke 2021). After conducting these six steps for each research stage independently, they were repeated throughout all the results collectively to generate the methodological framework.

## 7 Results and Discussion

The analysis generated a methodological framework that captures the enabling measures which supported meaningful participation throughout the study. These findings reflect the insights of participants, the contributions of the co‐researcher and the researcher's reflexive engagement within the field. Rather than focusing solely on barriers within the built environment, participants consistently highlighted the conditions that made the research process itself inclusive. These conditions became central to the development of the framework.

Across the four stages of data collection, participants shaped decisions about research settings, communication methods, pacing and the design of activities. The co‐researcher played a key role in identifying what made the research environment feel inclusive. Together, these insights formed the basis of the enabling measures presented below.

### 7.1 The Methodological Framework

The framework represented by Table 2, below, provides a summary of the enabling measures used in the study, highlighting key attributes contributing to the success/significance of each measure. In total, there are 15 enabling measures organised under three subgroups: logistical, technical and relational. All were implemented throughout the data collection period, ensuring the inclusivity of the research environment. They were co‐developed with the participants and the co‐researcher and informed by employment experience and previous literature.

**TABLE 2:** Summary of enabling measures used throughout the data collection.

Enabling measure	Success/significance attributes	Stage 1	Stage 2	Stage 3	Stage 4
Logistical					
1. Facilitation: Employing a disabled co‐researcher	− Conversation mediator − Accessibility officer − Engagement advisor − Recruiter − Tackle prejudice − Building rapport	√	√	√	√
2. Collaboration and employment: With a self‐advocacy organisation	− Enhanced recruitment − Building rapport − Deepened the understanding of the challenges faced by participants	√	√	√	√
3. Familiarity: Conducting activities in familiar locations	− Reduced stress − Facilitated comfort and openness in discussions	√	√		
4. Carers: Inclusion of support workers and carers	− Provided context for some insights − Provided support, especially for participants with lower physical mobility			√	√
5. Incentives: Offering non‐monetary incentives for participation	− Increases participant motivation − Participants felt valued	√	√	√	√
6. Resource adjustments: Extended time and budget	− Non‐rushed communication − Allowed the employment of the co‐researcher	√	√	√	√
Technical					
7. In‐person: Verbal admission and face‐to‐face interactions	− Facilitated a higher independence level for the participants − Building rapport − Allowed more observational space	√	√	√	√
8. Easy‐Read: All participant‐facing materials were in Easy‐Read format	− Enhanced comprehension and independence among participants	√	√	√	√
9. Prompts: Using cue cards, activities and photos	− Kept the research process interesting − Simplified the complex ideas − Aided memory − Created a more casual atmosphere	√	√	√	√
10. Open questions: Using open‐ended questions	− Allowed expressive depth − Participants felt that their opinions were valued	√	√	√	√
11. Compartmentalisation: Clustering different activities into different locations	− Organised and focused the workshop experiences − Supported fresh starts				√
12. Personalisation: Accommodating tasks based on the participant's abilities	− Made the research less formal and, consequently, more approachable − Enhanced engagement		√		√
Relational					
13. Empowerment: Prioritising participant voices and suggestions	− Enabled a higher independence level for the participants − Ensured their perspectives led the design of the activities	√	√	√	√
14. Reassurance: Continuous reassurance and empathy about privacy and anonymity	− Reduced stress − Fostered a supportive research environment − Participants felt less stressed − Encouraged openness, securing trust and deeper insights	√	√	√	√
15. Informality: Casual research interaction	− Enhanced engagement − Building rapport	√	√	√	√

#### 7.1.1 Logistical Measures

The logistical measures (nos. 1–6) reflect the practical conditions that enabled meaningful participation throughout the study. A central logistical measure was the employment of a co‐researcher with an intellectual disability from the outset of the project. Their involvement created an inclusive research environment and supported participants to share their insights more freely, which was evident in previous research (Gary et al. 2012). The co‐researcher's reflections illustrate the significance of this role:

…I shared experiences, e.g., the best times, days of the week, and best ways to communicate with members. I was also given the chance to tweak the words, e.g., the consent forms, or do an explanatory video format of what the project is all about. There were incentives such as certificates and a one‐off tea or coffee with cake or pastry at a café of the participant's choice as a thank‐you for their time!…

This emphasises the importance of their role in bridging the gap between research and lived experience. Additionally, it highlights how research can either facilitate or impede the participation of people with intellectual disabilities. Their position as an insider within the self‐advocacy organisation enabled them to bridge the gap between research and lived experience. They contributed to multiple aspects of the project, including:

- Addressing prejudice surrounding intellectual disability.
- Building rapport with participants.
- Advising on accessible communication.
- Facilitating recruitment.
- Supporting interviews and pilot studies.
- Guiding participants to venues.
- Assisting participants with limited motor skills during workshops.

These contributions demonstrate how the co‐researcher shaped the accessibility, relevance and relational quality of the research process, aligning with calls for research that redistributes power and centres lived experience (Walmsley 2001; Strnadová et al. 2016).

Collaboration with the self‐advocacy organisation was another key logistical measure. While initially intended to support recruitment, the partnership evolved into a sustained relationship that extended beyond formal fieldwork. This mirrors findings from disability research showing that long‐term collaboration strengthens rapport, trust and the authenticity of participant engagement (Walmsley 2001; Tuffrey‐Wijne and Butler 2010). Regular interactions outside formal sessions deepened the researcher's understanding of participants' everyday challenges, ensuring that the study remained grounded in real‐world experiences and aligned with the priorities of the intellectual disability community (Rojas Pernia and Haya Salmón 2021).

Conducting activities in familiar locations helped reduce anxiety and supported open discussion. When familiar settings were not feasible, the presence of support workers, family members or friends helped mitigate unfamiliarity. Support workers were included only in later stages to avoid overshadowing participants' voices, a concern raised in previous research (Sitbon and Farhin 2017). Their involvement provided contextual insights during focus groups and practical assistance during hands‐on co‐design activities, consistent with findings from Gaudion et al. (2015).

Non‐monetary incentives were used to recognise participants' time and contributions. Based on the co‐researcher's recommendations, participants received certificates and small tokens of appreciation, which helped them feel valued. Such incentives are recognised as ethical and effective in qualitative research, promoting participation without the concerns associated with monetary payments (Grant and Sugarman 2004; Head 2009). The co‐researcher, however, received financial compensation through formal employment, reflecting the depth and professionalism of their role and avoiding tokenism.

Inclusive participation required adjustments to both time and budget. Extending the project timeline allowed for unhurried communication, consistent with recommendations for inclusive research (Gary et al. 2012; Mencap 2016). Budget adjustments were also necessary to accommodate the employment of the co‐researcher and the additional time required for inclusive practices, reflecting broader findings that inclusive research typically requires greater resources and flexibility (Tuffrey‐Wijne and Butler 2010; Walmsley et al. 2018). While logistical measures established the practical foundations for inclusion, meaningful participation also depended on how research activities were designed and facilitated. The following section outlines the technical measures that supported communication, understanding and engagement.

#### 7.1.2 Technical Measures

The technical measures (nos. 7–12) describe how research activities were designed and facilitated to support communication, understanding and engagement. The primary technical measure was the use of verbal communication and in‐person interaction. Participants consistently responded more confidently and openly when activities were conducted face‐to‐face, which provided a wider observation space and supported rapport‐building. While written materials were rarely relied upon, Easy‐Read documents were made available for participants who wished to engage more deeply with the research. This approach reflects recommendations from organisations advocating for accessible communication formats (National Health Service England 2018; Mencap 2024); however, verbal interaction remained the most effective method for the participants.

**FIGURE 2:**
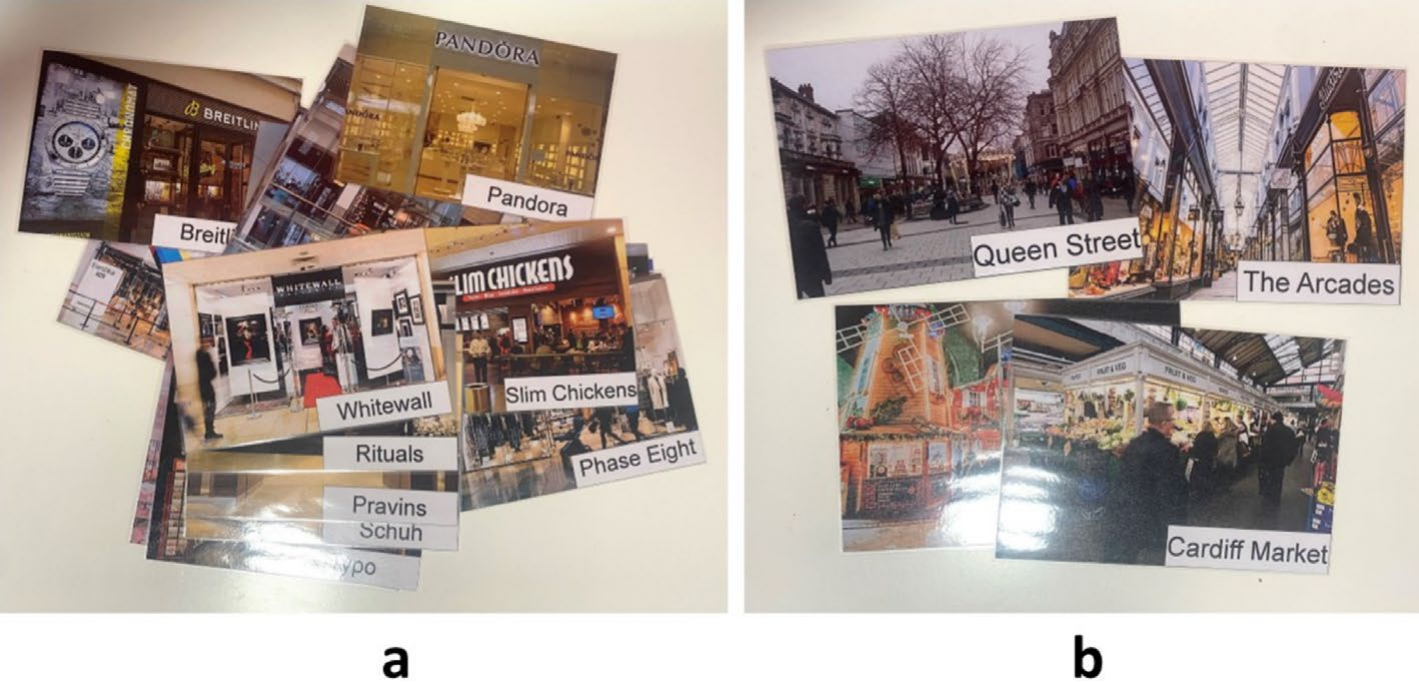
A sample of the cue cards used for Stage 2 (Exploring). (a) A random sample of the centre's shops for wayfinding activities; (b) shopping places/spaces around Cardiff to prompt conversation around preferences.

**FIGURE 3:**
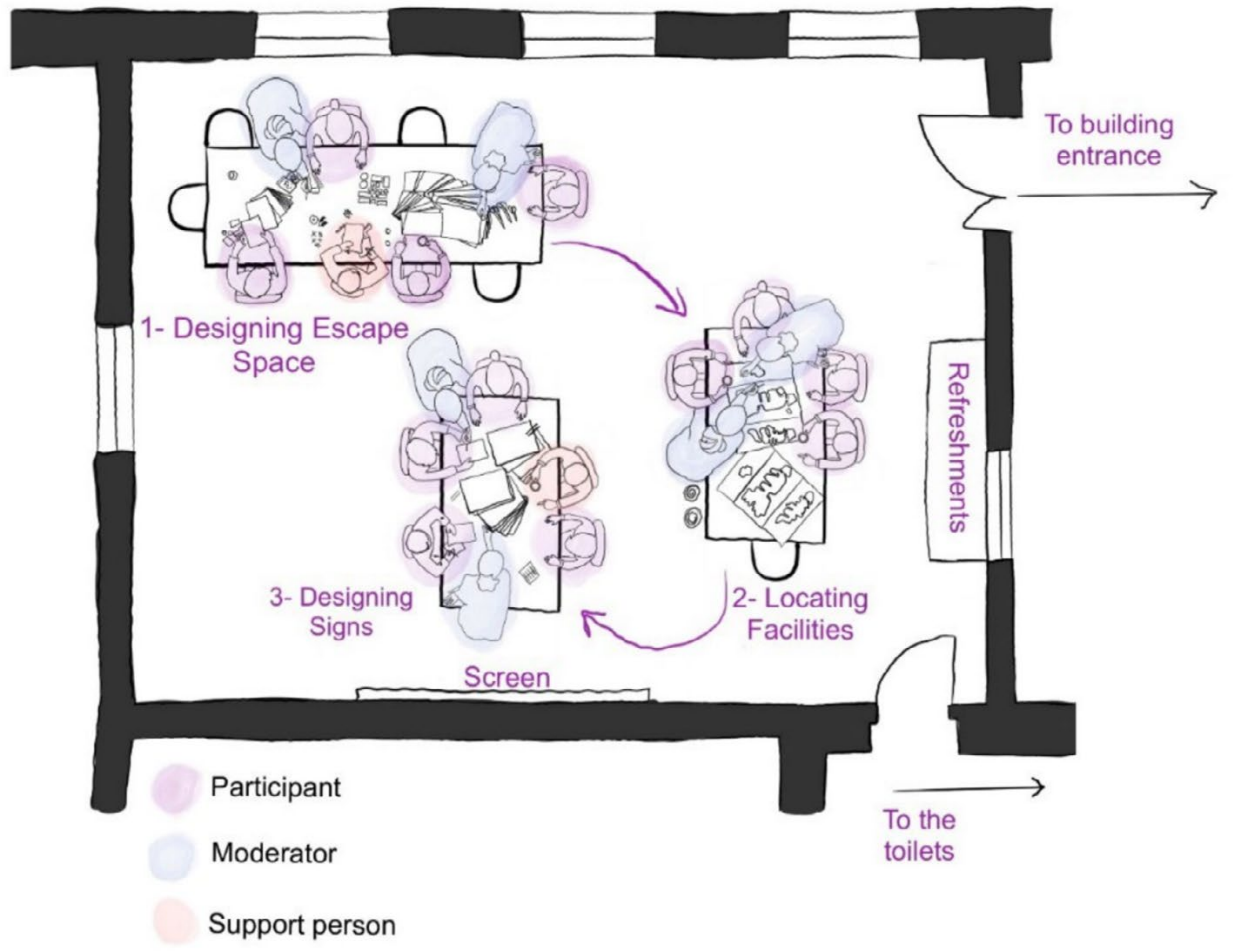
The furniture layout and room organisation for the co‐design workshop (Stage 4), with distinct zones/tables for each activity.

The use of various prompts, such as cue cards and activities, helped overcome the formality of the research, supported understanding of the task and aided memory (see Figure 2). This was inspired by the recommendations of organisations and previous research (Gaudion et al. 2015; Mencap 2016; Fudge Schormans et al. 2019). Another technical measure was using open questions, which allowed a bigger space for self‐expression and reflection, following Mencap's (2016) guidance on communicating with people with intellectual disabilities. Consequently, the participants felt that their perspectives were valued, as many people with intellectual disabilities relish the opportunity to be listened to (Gary et al. 2012). Another technical measure was compartmentalisation for the co‐design workshops, where each activity had its designated zone in the workshop space to minimise confusion, promote fresh starts and enhance focus (see Figure 3).

Lastly, there were different levels of personalisation and flexibility in all stages. For example, the recruitment process supported different contact methods according to participant preference. Moreover, the researcher was flexible about the participants' schedules. For example, some research activities were conducted on weekends, resulting in increased participation. Additionally, most research activities were designed to be adaptable, such as incorporating more breaks and allowing participants to choose their break locations during walking interviews. This aligns with recent findings that emphasise the need for flexibility in knowledge production with people with intellectual disabilities (Gjermestad et al. 2020; Rojas Pernia and Haya Salmón 2021).

While technical measures focused on how research activities were designed and facilitated, inclusion also depended on the relational and ethical stance taken towards participants. The following section outlines the relational measures that supported trust, respect and meaningful engagement.

#### 7.1.3 Relational Measures

Relational measures (nos. 13–15) describe the interpersonal and ethical conditions that supported trust, comfort and meaningful engagement throughout the study. Empowering the participants by prioritising their voices was a vital relational measure, especially during the latter stages when support workers were allowed to participate. Additionally, the design of the walking interviews depended on following the participants' lead in a personalised manner, maximising their contribution space. Moreover, both the researcher and the co‐researcher ensured continuous assurance regarding privacy and anonymity during the different stages. This was inspired by the co‐researcher and was highly appreciated by the participants.

The last relational measure was informality, which was supported by casual interactions outside of research activities due to employment, leading to building rapport with the community. As a result, many participants provided lengthy, detailed examples of their preferences and the barriers they encountered. This was also where the co‐researcher's role shone, as they reflected on their own experience, making it more relatable to the participants. The participants enjoyed long conversations during the breaks. Although those conversations might not have been directly related to the research topics, they helped build rapport between the researcher and participants, resulting in a substantial amount of qualitative data totalling over 1500 h of engagement, including employment. However, it is essential to acknowledge that dedicating this number of hours to participant engagement was made possible due to the unique privilege of having an employment position that facilitated continuous informal interactions, which may not be replicable for professional research teams with different resource constraints.

While this study did not fully meet all the criteria of inclusive research, it incorporated many of its core principles by extending the role of a co‐researcher with an intellectual disability beyond consultation and into meaningful involvement across multiple stages of the research process. Inclusive research exists along a continuum of participation, from consultative to fully co‐produced approaches (Strnadová et al. 2016), and this study sits within that continuum while acknowledging ongoing structural and institutional constraints.

These enabling measures, tailored to the needs and preferences of people with intellectual disabilities, offer a framework that can guide future architectural research. Researchers can create a more inclusive research environment by following these methodological considerations. This approach not only enhances the engagement and contribution of disabled participants but also ensures that the resulting architectural insights genuinely reflect their needs and experiences.

The framework demonstrated that inclusive architectural research depends not only on accessible tools but on the relational, temporal and organisational conditions that allow people with intellectual disabilities to participate meaningfully. They also highlight how everyday practices, such as informality, familiarity and sustained presence, can be as influential as formal methodological choices in shaping the quality of engagement.

Recommendations for future research include allocating sufficient time for relationship‐building, involving co‐researchers with lived experience from the outset, and designing flexible, multimodal activities that can be adapted to different communication preferences. Strengthening partnerships with self‐advocacy organisations and embedding informal, trust‐based interactions can further enhance the authenticity and depth of participation.

## 8 Conclusion

This article has presented a methodological framework designed to support inclusive architectural research with people with intellectual disabilities. The framework brings together logistical, technical and relational enabling measures that shaped an accessible and empowering research environment. These measures ensured that participant voices were prioritised throughout the study and that the research process reflected their preferences, communication styles and lived experiences.

The findings demonstrate that inclusive architectural research requires more than accessible methods; it requires deliberate attention to relationships, resources and the redistribution of power. By centring the expertise of people with intellectual disabilities, the framework challenges traditional research hierarchies and contributes to wider debates on disability, participation and epistemic justice. It also highlights the practical realities of inclusive research, including the need for flexibility, extended timelines and resource adjustments to support meaningful involvement.

Although this study was situated within a specific context in Cardiff, enabled and shaped by the constraints and opportunities inherent in doctoral research, its core principles are transferable to other settings, situations and disciplines. Researchers are encouraged to adapt, test and refine this framework to move closer to genuine co‐production and shared ownership of research processes. Embedding such inclusive methodologies within architectural practice and research has the potential to produce environments that are not only accessible but genuinely reflective of the diverse communities they serve.

At its heart, this work recognises people with intellectual disabilities as overlooked experts in the built environment and demonstrates how their involvement can lead to richer, fairer and more inclusive research outcomes.

The Supporting Information includes the full interview schedules, walking‐interview prompts, focus‐group agendas and co‐design workshop materials used in the study. These resources are provided to support transparency, replicability and practical uptake by other researchers conducting inclusive or participatory research with people with intellectual disabilities.

## Funding

This article stems from a doctoral study funded by a full scholarship from the Ministry of Higher Education of the Arab Republic of Egypt.

## Ethics Statement

The Research Ethics Committee at the Welsh School of Architecture, Cardiff University, granted ethical approval for the study (SREC reference: 2204, 22,111 and 23,048).

## Supporting information


**Figure S1:** A sample of the cue cards used during the semi‐structured interviews.
**Figure S2:** The pilot interview route.
**Figure S3:** One of the shopping centre's entrances featuring multiple doors without labels.
**Figure S4:** Walking interview structure.
**Figure S5:** A sample of the cue cards used for the walking interviews. (a) A random sample of the centre's shops for wayfinding activities, (b) Shopping places/spaces around Cardiff to prompt conversation around preferences.
**Table S1:** Interview questions for frequent users and their source.
**Figure S6:** The interview route for the non‐frequent users, where questions are strategically localised to correspond with the questions being asked. It stops at question 15, as the wayfinding activities begin from question 16, and consequently, the route will follow the participant's lead.
**Table S2:** The agenda of the focus groups.
**Figure S7:** The focus group presentation was aided by photos of the shopping centre.
**Figure S8:** Introductory and eligibility questions form.
**Figure S9:** The workshops' presentation was aided by prompt photos.
**Figure S10:** The map used to locate recovery spaces with the existing facilities and landmark shops (brand names anonymised for confidentiality).
**Figure S11:** Recovery space base model.
**Figure S12:** Wall colour selection.
**Figure S13:** Floors selection.
**Figure S14:** Openings selection.
**Figure S15:** Furniture options.
**Figure S16:** An example of the centre maps provided to participants with existing seats, signposted by facilities and landmark shops, which the participants used to locate extra seats where needed (brand names anonymised for confidentiality).
**Figure S17:** Signs types for the participants to choose from.
F**igure S18:** Coloured papers and pens for sign designing sign.
**Figure S19:** Welsh translation for common signs.

## Data Availability

The data that support the findings of this study are available on request from the corresponding author. The data are not publicly available due to privacy or ethical restrictions.
