# Supplementary material for: (En)Abling Architectural Research: Co‐Designing With People With Intellectual Disabilities
Source: J Appl Res Intellect Disabil. 2026 Jul 1;39(4):e70275. doi: 10.1111/jar.70275 (PMC13320296; doi:10.1111/jar.70275)
Supplement: Supplementary file 1 — Figure S1: A sample of the cue cards used during the semi‐structured interviews. Figure S2: The pilot interview route. Figure S3: One of the shopping centre's entrances featuring multiple doors without labels. Figure S4: Walking interview structure. Figure S5: A sample of the cue cards used for the walking interviews. (a) A random sample of the centre's shops for wayfinding activities, (b) Shopping places/spaces around Cardiff to prompt conversation around preferences. Table S1: Interview questions for frequent users and their source. Figure S6: The interview route for the non‐frequent users, where questions are strategically localised to correspond with the questions being asked. It stops at question 15, as the wayfinding activities begin from question 16, and consequently, the route will follow the participant's lead. Table S2: The agenda of the focus groups. Figure S7: The focus group presentation was aided by photos of the shopping centre. Figure S8: Introductory and eligibility questions form. Figure S9: The workshops' presentation was aided by prompt photos. Figure S10: The map used to locate recovery spaces with the existing facilities and landmark shops (brand names anonymised for confidentiality). Figure S11: Recovery space base model. Figure S12: Wall colour selection. Figure S13: Floors selection. Figure S14: Openings selection. Figure S15: Furniture options. Figure S16: An example of the centre maps provided to participants with existing seats, signposted by facilities and landmark shops, which the participants used to locate extra seats where needed (brand names anonymised for confidentiality). Figure S17: Signs types for the participants to choose from. Figure S18: Coloured papers and pens for sign designing sign. Figure S19: Welsh translation for common signs. [file JAR-39-e70275-s001.docx]

# Supplementary material for (En)Abling Architectural Research: co-designing with people with intellectual disabilities

This supplementary file provides the full interview schedules, walking‑interview structure, focus‑group agenda, and co‑design workshop materials used in the study. These tools were developed through pilot testing, context familiarisation, and collaboration with a self‑advocacy organisation and a co‑researcher with lived experience of intellectual disability. They are included to support transparency, replicability, and practical uptake by other researchers conducting inclusive or participatory research with people with intellectual disabilities.

## Interview Questions

The interviews were kept concise and straightforward, adhering to literature review. They were divided into two parts:

1. **Personal and Eligibility Questions**
   The first part of the interview consisted of personal and eligibility questions, including three mandatory and two optional questions. These questions were designed to collect fundamental demographic information while ensuring that participants met the study's eligibility criteria. Mandatory questions are marked with an asterisk (*).
   1. How old are you? *
   2. How would you describe your gender?
   3. Do you have an intellectual disability? *
   4. What is your intellectual disability?
   5. Do you live in Cardiff? * If no, do you visit Cardiff?
2. **Experience with Buildings**
   The second part of the interview focused on participants’ experiences with different buildings in Cardiff. This section consisted of seven questions, of which six were open-ended, allowing participants to share more detailed information about their interactions with the various buildings around Cardiff.
   1. Do you study, work, both, or neither?
   2. What building(s) are you based in during daytime hours?
   3. What other building(s) do you visit on a day-to-day basis?
   4. What other building(s) do you visit on weekends or holidays?
   5. Do you have a favourite public building(s) that make you feel at ease? If yes, which buildings are they?
   6. Do you avoid certain buildings because you don’t feel comfortable in them? If yes, which buildings?
   7. Are there any further comments you would like to add?

Given that the interview questions focused on a range of buildings across Cardiff, it was not feasible to accompany each participant to all these locations. As a result, participants may struggle to recall their experiences or recognise buildings from the interview questions alone. To help participants remember their experience with buildings, cue cards displaying images of different building types across Cardiff were utilised, inspired by the aforementioned recommendations (see Supplementary Figure 1). These cue cards served as prompts and memory aids for participants who either did not recall their experiences or were uncertain about how to respond.


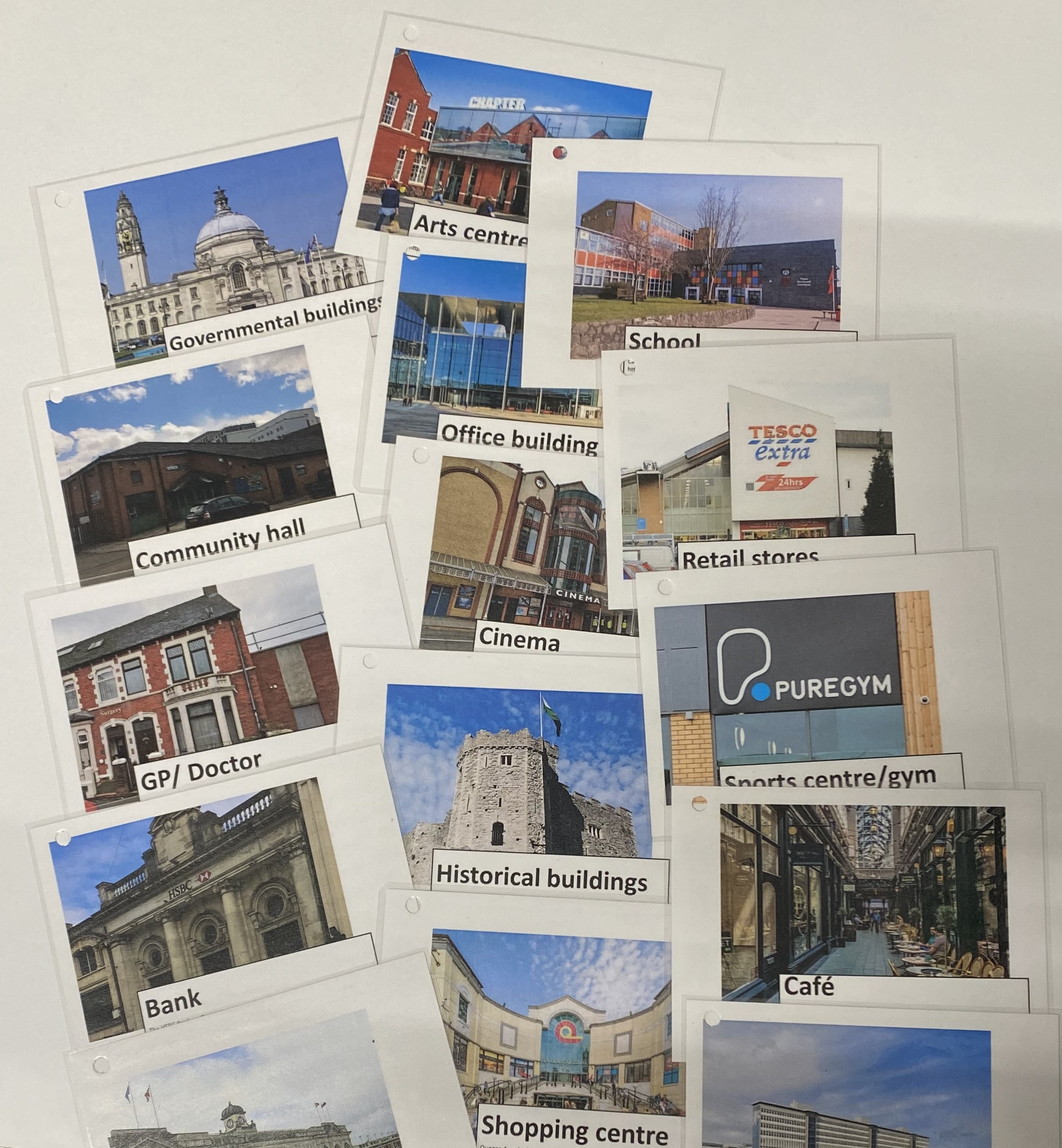


Supplementary Figure 1: A sample of the cue cards used during the semi-structured interviews.

## Walking Interview structure

This research stage aimed to answer the following questions:

- How do people with an intellectual disability use the shopping centre?
- What makes a shopping centre accessible for people with an intellectual disability?
- What barriers do people with an intellectual disability face in a shopping centre?
- How do different architectural elements in a shopping centre affect the experiences of people with an intellectual disability?

To develop the walking interview questions, a pilot walking interview was conducted with the facilitator to assess their accessibility, identify new themes, and investigate any technical issues with the interview, such as its length or tiring nature. The questions were inspired by the barriers found in the literature for people with an intellectual disability and the literature on the design considerations for shopping centres.

The pilot interview lasted around 75 minutes and included twelve questions to explore the shopping centre's general advantages and disadvantages. Some questions were asked while walking around the centre, while others were asked after the walk. The interview was recorded on a password-protected mobile device, which were immediately uploaded to the university’s secure cloud storage. Once the successful transfer was confirmed, the original was deleted from the phone. Additionally, observations were made during the walk, and field notes were recorded.

1. **Questions during the walk**
2. Where do you usually enter the shopping centre from? And why?
3. What is your favourite shop in the shopping centre/ Where do you usually go to the shopping centre/ Where do you like to go inside the shopping centre?
4. What do you think about the lighting? Is it too bright or too dark? Do you like them?
5. Are you bothered by the smell, for example, walking by a perfume shop or a restaurant?
6. What about the crowd and the noise?
7. What do you think about the navigation and the wayfinding?
8. What do you do if you want to reach a specific shop but do not know where it is?
9. Are you OK with this shopping centre?
10. **Questions after the walk**
11. What do you hate the most about the shopping centre, or what would you change if you were changing anything?
12. Are you bothered that the shopping centre shifts from inside to outside?
13. Do any of your friends shop here?
14. What do you like best about this shopping centre?

The pilot interview was transcribed, and the interview route was mapped (see Supplementary Figure 2). The following barriers were identified through the analysis, which were echoed through observations during the employment role:

- **Wayfinding and navigation:** Barriers included vague navigation and unclear and inadequate signs.
- **Facilities:** The facilitator highlighted that the number of toilet locations and cubicles inside the toilets is insufficient. For example, the centre only has one toilet on the upper floor.
- **Entrance:** The entrance features numerous doors, making it somewhat confusing to determine which one to use for entry or exit. Each entrance or exit comprises several doors, varying between automatic and manual, with no indication of which is for entering and which for exiting (see Supplementary Figure 3), echoing Daisy’s comment mentioned in the previous chapter.


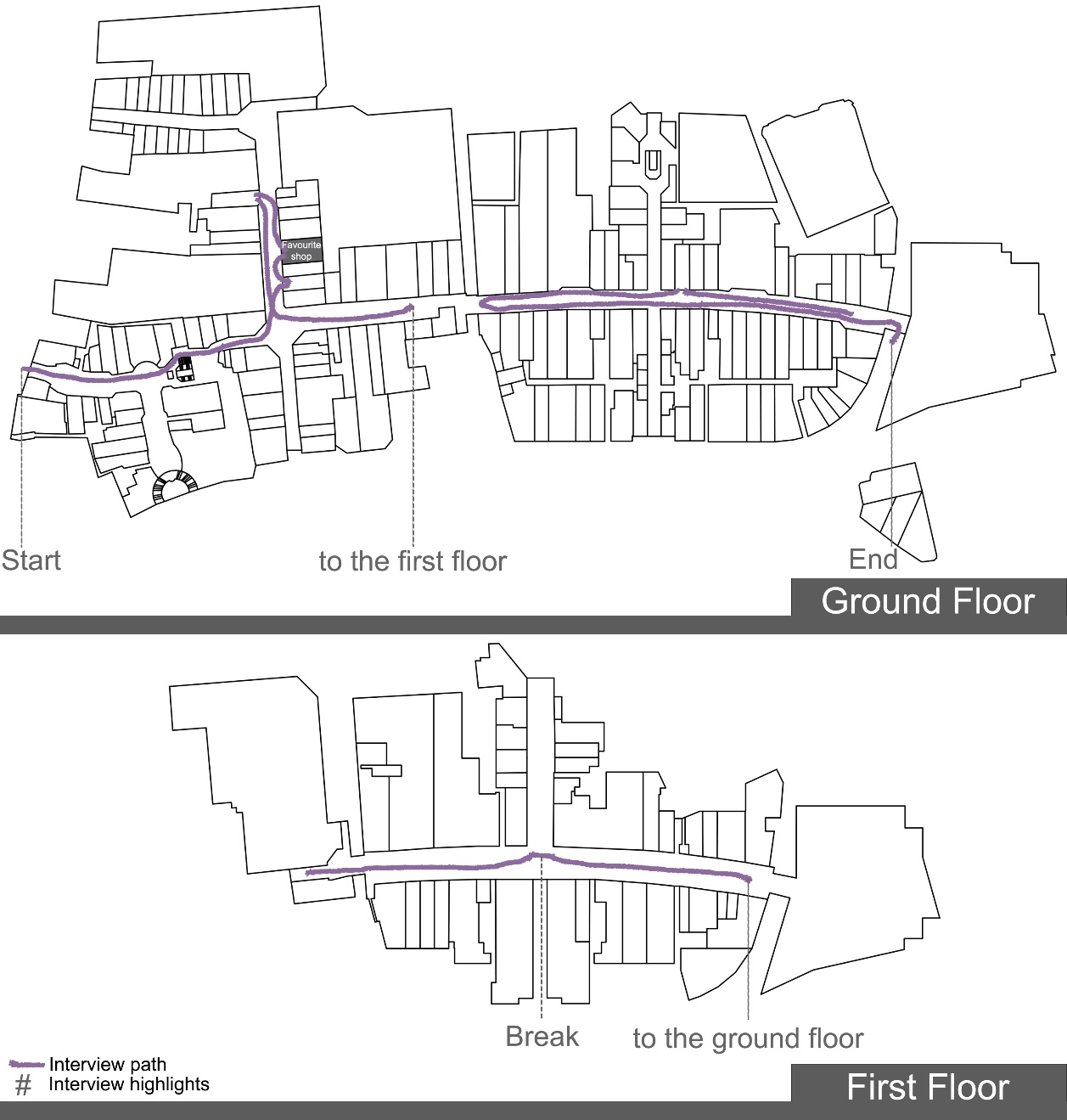


Supplementary Figure 2: The pilot interview route


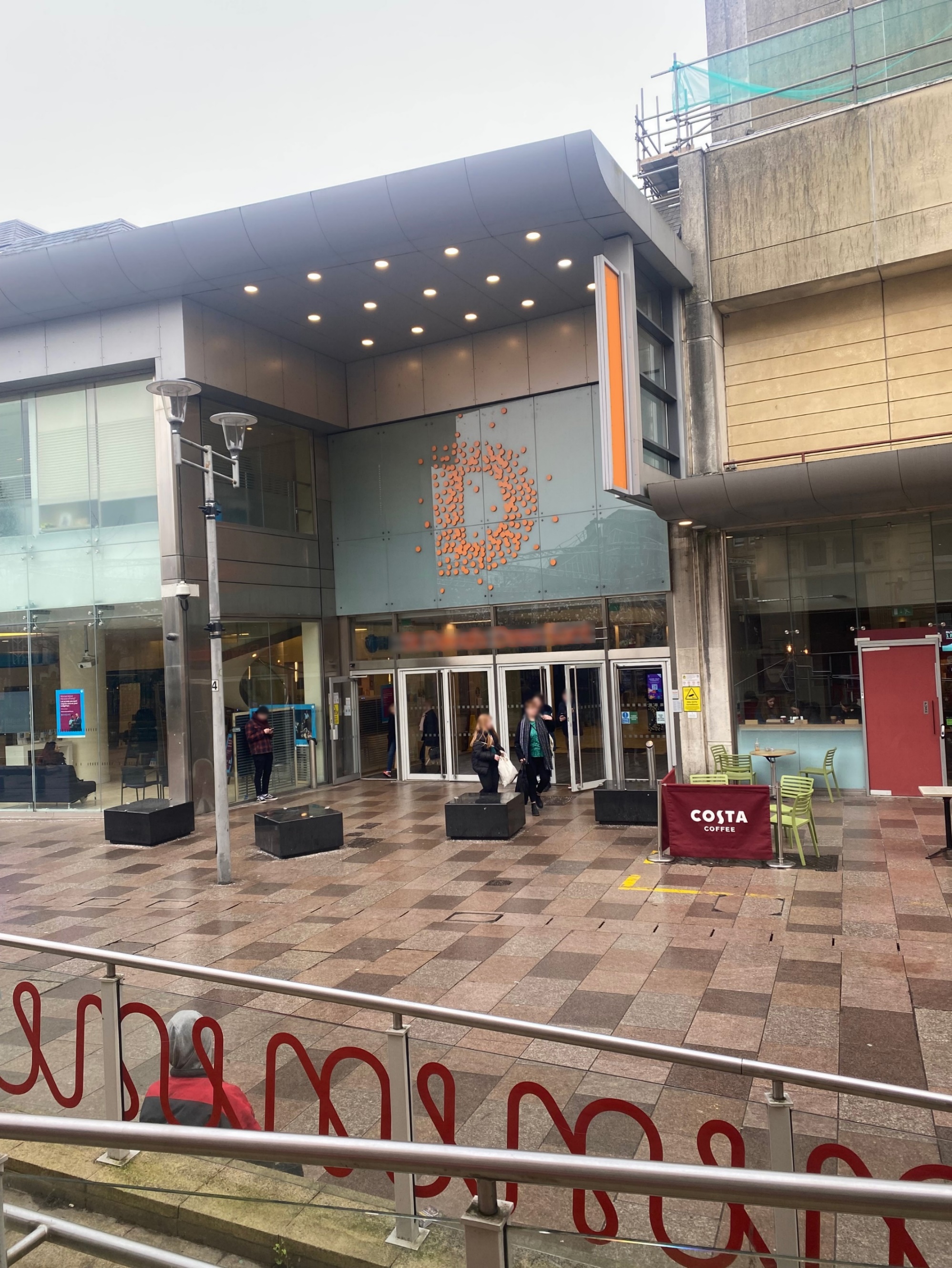


Supplementary Figure 3: One of the shopping centre’s entrances featuring multiple doors without labels

The pilot interview also discussed the centre's sensory environment, which the facilitator had no problem with. However, some navigational tasks made it clear that it is not easy to get around the centre. In addition, although the facilitator is a frequent visitor, she was unaware of the centre’s accessibility features, such as the hidden disability lanyard, autism-friendly services, the guide for autistic guests, the quiet room mentioned in the guide, or interactive maps.

### Interview question design

Following the analysis of the pilot interview, the interview questions were developed. There were two versions of the interview questions: i) for frequent visitors who visit the centre once a month or more, and ii) for first-time and non-frequent visitors who visit the centre less than once a month. Following the findings from the previous research stage, which indicated that familiarity influences how people with an intellectual disability perceive buildings, both versions maintained a similar structure:

1. **Eligibility questions and demographic questions:**

The first part of the interview included eligibility and demographic questions, with four mandatory and one optional question. These questions were designed to gather basic demographic information while ensuring that participants met the study's eligibility criteria. The last question in this section asked about the frequency of visiting the centre to decide the structure of the interview. The questions were as follows, noting that mandatory questions are marked with *:

1. How old are you? *
2. Are you male/female/other?
3. Do you have an intellectual disability? *
4. Do you live in Cardiff? If the answer is no, do you visit Cardiff? *
5. How often do you go to St. David’s shopping centre? *
   1. Once a month or more
   2. Less than once a month
6. **Questions during walking around the centre:**This part of the interview included 33 questions for frequent visitors and 28 questions for non-frequent visitors, where some questions about preferences were removed. In both scenarios, the questions were divided into five categories. preferences, sensory environment, centre facilities, wayfinding, navigation and department stores (see Supplementary Figure 4). The questions in this part and the following one were inspired by the findings of previous literature on accessibility for people with an intellectual disability, previously reviewed in Chapter 2, the findings of stage 1, the literature on shopping centres, context familiarisation, pilot study, and employment experience (see Supplementary Table 1).

It aimed to capture the participants' experience within the shopping centre and the impact of specific architectural features on their accessibility. It included locational questions, which are questions related to the surroundings, such as questions about lighting, specifically when pointing to a light source or skylight. It also included wayfinding activities to observe how participants navigated the buildings, in addition to the utilisation of cue cards (see Supplementary Figure 5 a).

1. **Questions after the walk:**

Considering the length of the interview and to enhance accessibility, after completing the first set of questions, a break was taken at a location of the participants’ choice, where refreshments were provided. The second set of questions was addressed in the same location as the break. This part had 18 questions in both scenarios, divided into three categories: preferences, the centre’s accessibility features and wayfinding and navigation (see Supplementary Figure 4). It aimed to understand the building experience holistically and reflect on the walking interview. Cue cards were also utilised in this section (see Supplementary Figure 5 b).

Locational questions

Locational and cue cards questions

Questions with cue cards

Supplementary Figure 4: Walking interview structure


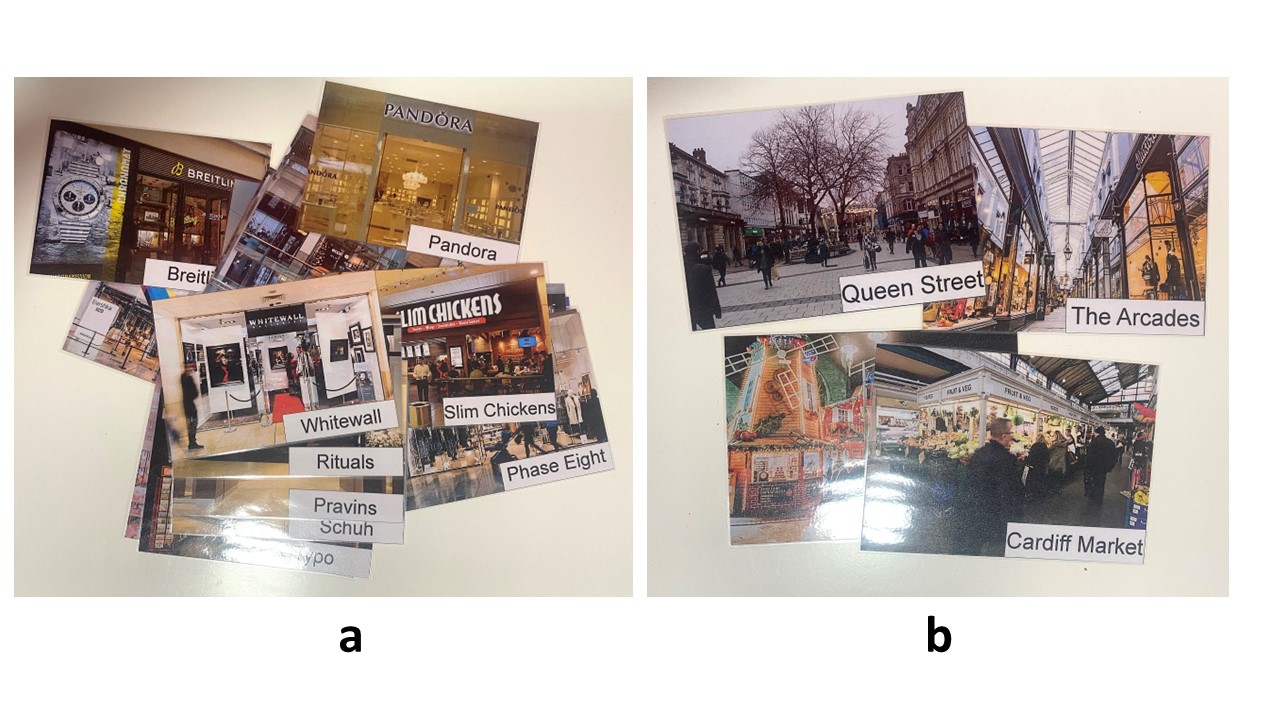


Supplementary Figure 5: A sample of the cue cards used for the walking interviews. a) A random sample of the centre’s shops for wayfinding activities, b) Shopping places/spaces around Cardiff to prompt conversation around preferences

Supplementary Table 1: Interview questions for frequent users and their source

| Question | Intellectual disability accessibility literature | Stage 1 findings | Shopping centres literature | | Context familiarisation | | | Pilot study | Employment experience | |
| --- | --- | --- | --- | --- | --- | --- | --- | --- | --- | --- |
| **During the walk** | | | | | | | | | | |
| Preferences | | | | | | | | | | |
| 1. Where do you want to start? |  | √ | |  | |  |  | | | √ |
| 2. Do you think the entrance is clear enough? |  | √ | |  | |  | √ | | | √ |
| 3. What is your favourite shop here? Can you take us there? | √ | √ | |  | |  | √ | | | √ |
| Sensory environment | | | | | | | | | | |
| 4. Do you like the lighting here? | √ |  | | √ | |  |  | | |  |
| 5. Do you like these [occasional] decorations? | √ |  | |  | |  |  | | | √ |
| 6. Is it smelly here? If yes, does it bother you? | √ |  | | √ | |  |  | | |  |
| 7. Do you think it is noisy? If yes, does it bother you? | √ |  | | √ | |  |  | | |  |
| 8. Do you find it crowded here? If yes, does it bother you? |  |  | | √ | |  |  | | | √ |
| 9. Do you like the materials of floors, ceilings and walls? |  |  | | √ | |  |  | | | √ |
| 10. Do you like the colours of the walls, floors, and ceiling? |  |  | | √ | |  |  | | | √ |
| Centre facilities |  |  | |  | |  |  | | |  |
| 11. Can you take us to your favourite resting space? | √ |  | | √ | |  | √ | | | √ |
| 12. Why do you like it? |  |  | | √ | |  |  | | | √ |
| 13. Do you think there is enough resting space here? Why? |  |  | | √ | |  |  | | | √ |
| 14. Do you know where the toilets are? Can you take us there? | √ |  | | √ | |  | √ | | | √ |
| 15. Do you think it is easy to find toilets? | √ |  | | √ | |  | √ | | | √ |
| 16. Do you think there are enough toilet locations? |  |  | | √ | |  | √ | | |  |
| 17. Do you think there are enough cubicles inside the toilet? |  |  | | √ | |  | √ | | |  |
| Wayfinding and Navigation |  |  | |  | |  |  | | |  |
| 18. Do you like that the centre is open and double height? Does it help you locate things better? Does it feel noisy or intimidating? |  | √ | | √ | |  |  | | | √ |

| Question | Intellectual disability accessibility literature | Stage 1 findings | Shopping centres literature | Context familiarisation | Pilot study | Employment experience |
| --- | --- | --- | --- | --- | --- | --- |
| 19. Do you think you can move easily between floors? |  | √ | √ |  | √ | √ |
| 20. Do you think the signs are clear? Why? | √ | √ |  |  | √ | √ |
| 21. Do you know/use any shortcuts here? Can you show it to us? | √ |  |  |  |  | √ |
| **22. Activity 1:** The participant is shown pictures of random shops in the centre. They choose one they have not been to and then try to find it. | √ | √ |  |  | √ | √ |
| **23. Activity 2:** The participant is shown pictures of random shops in the centre. They choose one that they have not been to and then try to find it using interactive maps around the centre. | √ | √ |  | √ | √ | √ |
| Department stores |  |  |  |  |  |  |
| 24. Do you have a favourite department store [Department stores’ names]? Can you take us there? | √ |  | √ |  | √ | √ |
| 25. Are you confident in coming here alone and going shopping? Why? |  | √ |  |  |  | √ |
| 26. Do you like the lighting here? | √ |  | √ |  |  |  |
| 27. Is it smelly here? If yes, does it bother you? | √ |  | √ |  |  |  |
| 28. Do you think it is noisy? If yes, does it bother you? | √ |  | √ |  |  | √ |
| 29. Do you find it crowded here? If yes, does it bother you? | √ |  | √ |  |  | √ |
| 30. Do you like the materials? |  | √ |  |  |  | √ |
| 31. Do you like the colours of the walls, floors, and ceiling? |  | √ | √ |  |  |  |
| 32. Do you think it is easy to find what you need here? Why? | √ |  |  |  | √ |  |
| 33. Do you think the signs are clear enough? Why? | √ | √ |  |  | √ | √ |
| **After the walk** |  |  |  |  |  |  |
| Preferences |  |  |  |  |  |  |
| 1. Do you prefer this shopping centre or open centres/ shopping streets? Why? |  | √ |  |  |  | √ |
| 2. How often do you come here? |  | √ |  |  |  | √ |
| 3. How do you spend your time here? / Why do you come here usually? |  | √ |  |  |  | √ |
|  |  |  |  |  |  |  |
| Question | Intellectual disability accessibility literature | Stage 1 findings | Shopping centres literature | Context familiarisation | Pilot study | Employment experience |
| 4. Do you like shopping here? |  | √ |  |  |  | √ |
| 5. What is your favourite thing about this centre? |  | √ |  |  |  | √ |
| 6. Do you think other people with an intellectual disability might like this centre? Why? |  | √ |  |  |  | √ |
| 7. What do you hate about this centre? |  | √ |  |  |  | √ |
| 8. If you can change/add anything here, what would you change? | √ |  | √ |  |  | √ |
| 9. Are you confident in being here alone? Why? |  | √ |  |  |  | √ |
| 10. When you first came here, do you remember something that was inaccessible then and is fixed now, or have you gotten used to it now? |  |  |  |  | √ | √ |
| 11. What advice would you give an architect/designer of shopping centres? |  |  | √ |  |  | √ |
| Wayfinding and navigation |  |  |  |  |  |  |
| 12. Do you think it is easy to get around the centre? | √ | √ |  |  | √ | √ |
| 13. What do you think about the centre moving from inside to outside? |  | √ |  |  | √ | √ |
| 14. Do you know any shortcuts here? | √ | √ |  |  | √ | √ |
| 15. Can you give me directions from where we are seated now to the shop we visited earlier? | √ | √ |  |  | √ | √ |
| 16. Did you know that the centre offers assistance to hidden disabilities through the reception desk and a quiet room if you get overwhelmed? If the answer is no, now that you know it, would you consider using it? |  |  |  | √ |  | √ |
| 17. The centre has a brochure to support autistic users. What do you think of this brochure? Is it of any benefit to you? Why? |  |  |  | √ |  | √ |
| 18. Do you use different clues/landmarks in the centre to help you get around? If yes, what are these landmarks/ clues? | √ | √ |  |  | √ | √ |

The interview route depended on the participant’s familiarity with the centre. A route was partially designed for non-frequent visitors (see Supplementary Figure 6). While there was no pre-determined route for frequent visitors, the participants led the route.


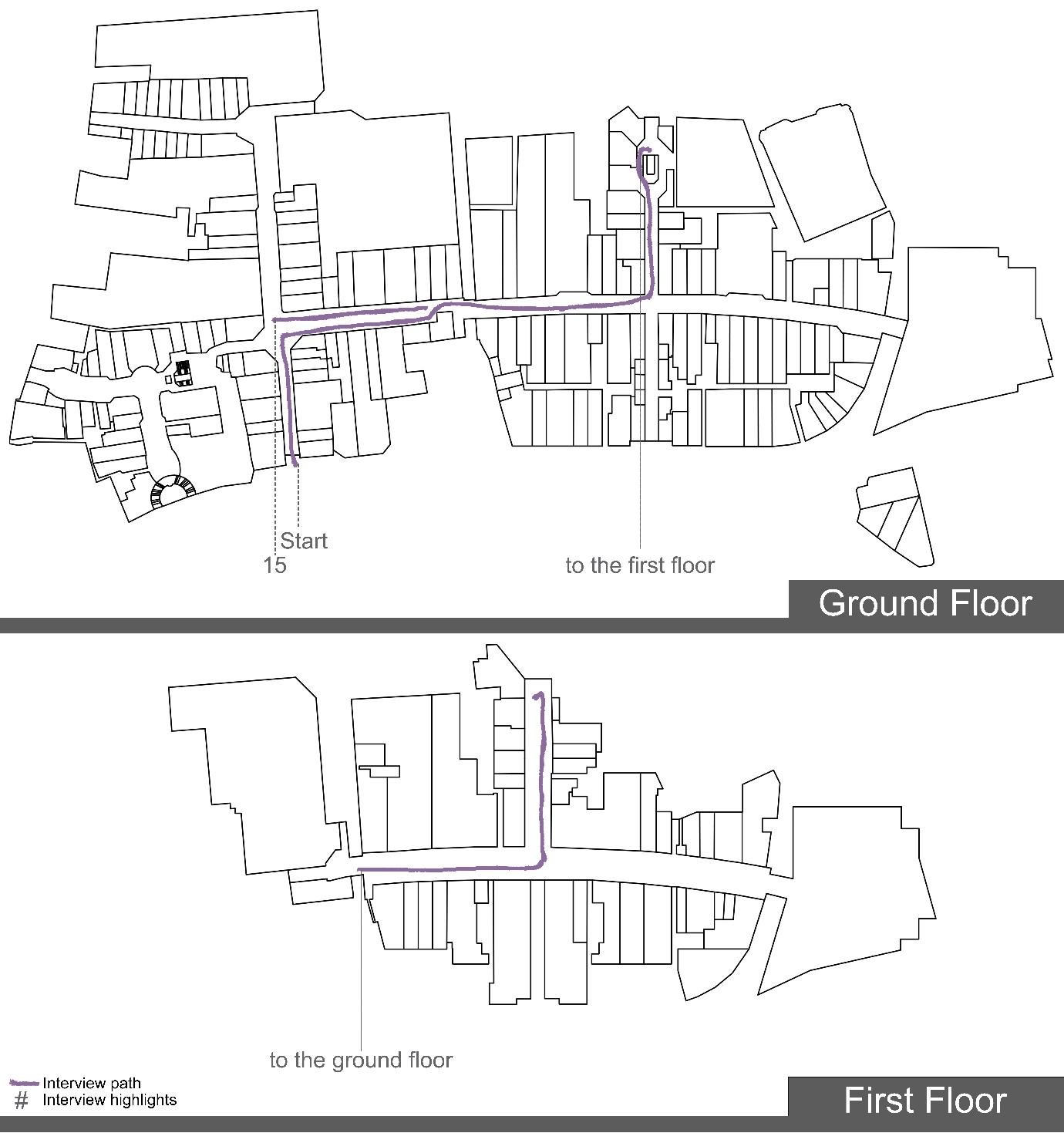


Supplementary Figure 6: The interview route for the non-frequent users, where questions are strategically localised to correspond with the questions being asked. It stops at question 15, as the wayfinding activities begin from question 16, and consequently, the route will follow the participant's lead

## Focus group structure

The focus groups aimed to verify and synthesise the architectural insights raised in the previous stages, to inform the agenda of the co-design workshops. It aimed to answer the following questions:

- What barriers do people with an intellectual disability face in shopping centres?
- How do different architectural elements affect the experiences of people with an intellectual disability?

The agenda included the themes that emerged previously from the walking interviews to verify them (see Supplementary Table 2). As it primarily aimed to verify the tangible, the intangible insights were not included in the agenda. The agenda was 170 minutes long, with each topic allocated 15 minutes for discussion, in addition to two breaks.

Supplementary Table 2: The agenda of the focus groups

| **Duration** | **Topics** | **Tools** |
| --- | --- | --- |
| 15 minutes | Introduction (workshop rules, project information sheet and consent) | Photo aid/ verbal admission |
| 15 minutes | Discussing Familiarity | Photo aid/ verbal admission/ voting |
|  | - If you are familiar with a place, will that make it more accessible to you? - Can you tell us how? - If you are going to a new place, what might help with familiarity? - What does familiarity mean to you in a shopping centre? (voting task)   - Familiar signs   - Familiar toilet locations   - Familiar stores/shops   - Familiar brands   - Familiar merchandise locations in the shop   - Familiar shop/store design (for example, colours)   - Familiar Staff   - Other | |
| 15 minutes | Discussing escape spaces | Photo aid/ verbal admission |
|  | - Do you need an escape space/quiet room/ sensory room in a shopping centre? Why? - If there is no space for a room, is there an alternative? | |
| 15 minutes | Comfort break |  |

| **Duration** | **Topics** | **Tools** |
| --- | --- | --- |
| 15 minutes | Discussing centre facilities | Photo aid/ verbal admission/ voting |
|  | - Vote for the facilities you need in a shopping centre to make it more accessible to you.   - Toilets   - Seats   - Restaurants and café   - Escape spaces/quiet rooms   - Maps   - Security staff   - Security cameras   - Safe materials (non-slippery floors)   - Staff   - Other…. - Does having different options in the shopping centre make it more accessible to you? For example: escalators, elevators and stairs? - What are other facilities options you think you need? | |
| 15 minutes | Discussing wayfinding | Photo aid/ verbal admission |
|  | - Do you use signs to help you get around the centre? - How important are signs in supporting your wayfinding? - What else would use to help you get around the centre? | |
| 30 minutes | Lunch Break |  |
| 15 minutes | Discussing space design | Photo aid/ verbal admission |
|  | - Do you prefer open and double-height places? Why? - How do you feel about crowds? - What is your coping mechanism with crowds? - How can an entrance be accessible? - What is an example of a good entrance? | |
| 15 minutes | Discussing user preferences | Photo aid/ verbal admission |
|  | - Do you prefer natural lighting? Why? - Does artificial lighting stress you? Why? - Do you prefer vivid colours more than neutral colours? Why? - Do you prefer decorative elements in the space? Why? | |
| 10 minutes | Voting | verbal admission/ voting |
| 10 minutes | Wrapping up (introducing the co-design workshops, signing up for the co-design workshops) | Photo aid/ verbal admission |

Being present in the relevant environment proved to be a significant asset during the previous stage, as it provided a valuable prompt for many participants and elicited numerous insightful comments. However, since the workshops could not be held at the shopping centre, considering the number of participants, distractions, lack of appropriate space and valuing the participants' privacy, this was compensated by using photos from the centre in the focus group presentation (see Supplementary Figure 7). Another prompt utilised in this stage was voting, based on the facilitator's recommendation, employment experience and previous research (Gary et al., 2012). Voting was utilised three times in the focus groups to enhance engagement (see Supplementary Table 2).


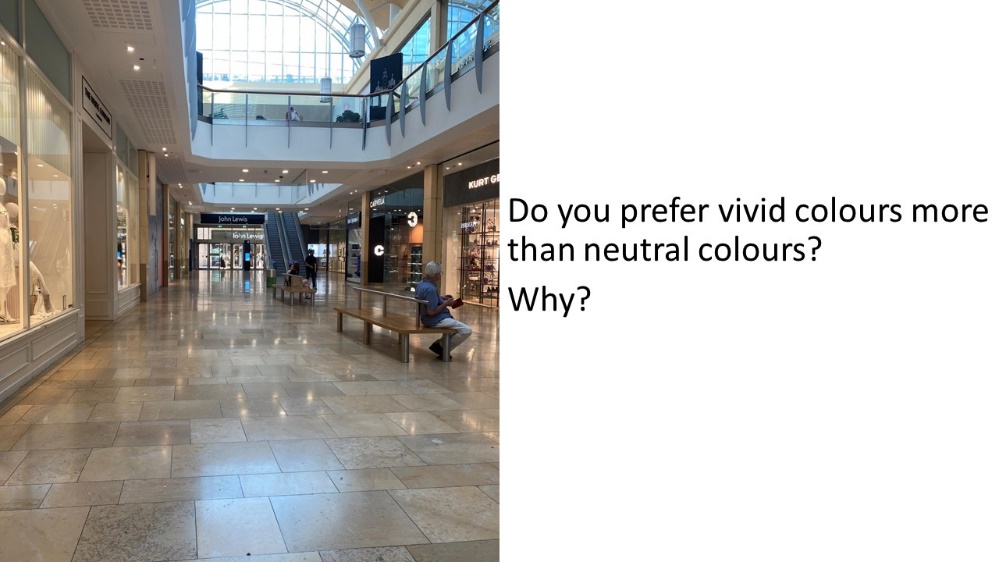


Supplementary Figure 7: The focus group presentation was aided by photos of the shopping centre

Following the same approach as the previous research stages, the project information sheet and consent form were prepared in Easy-Read format, primarily for the participants’ records. However, the participant information sheet was not prepared in a video format, as both the consent and the participant information sheet were admitted verbally as part of the focus group presentation. In addition, an introductory question sheet was also prepared in Easy-Read format to collect general information about the participants and ensure their eligibility before the start of the discussion (see Supplementary Figure 8).


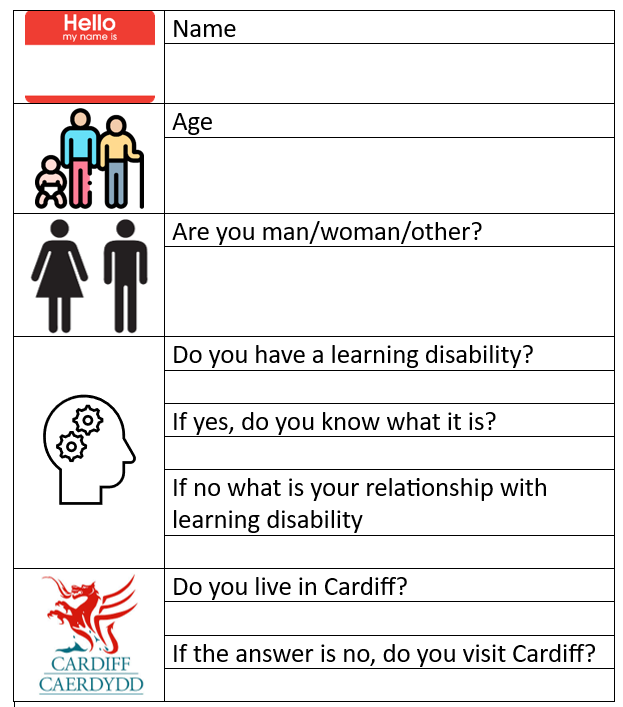


Supplementary Figure 8: Introductory and eligibility questions form

## Workshops structure

The workshops aimed to co-design solutions to the challenges raised in the previous research stages. Consequently, answer the final research question: What design strategies can make buildings more inclusive for people with an intellectual disability? Notably, previous research stages also contributed to answering these questions, as the participants recommended different strategies to overcome accessibility barriers.

##### Designing a recovery space

The first activity was to design a three-dimensional model of a recovery space individually. Since the design process can be challenging for non-designers and people with an intellectual disability, the design activity was translated into prompt questions, each of which included various tools and materials. These questions were added to a presentation aided with photos as additional prompts (see Supplementary Figure 9).


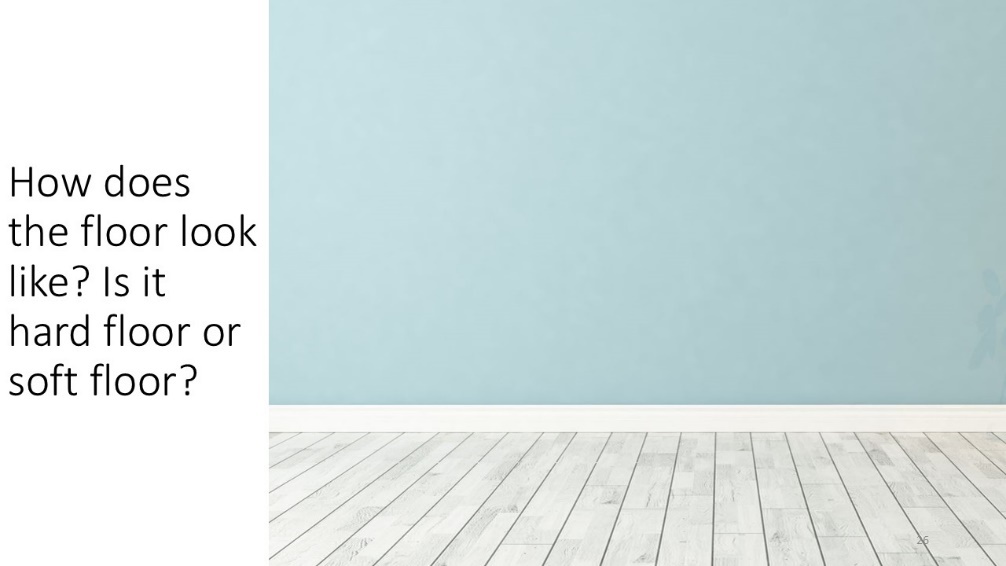


Supplementary Figure 9: The workshops’ presentation was aided by prompt photos

1. **Where is your recovery space?**

Participants were provided with the centre map in large print with the location of main facilities and landmark shops, informed by the walking interviews (see Supplementary Figure 10).


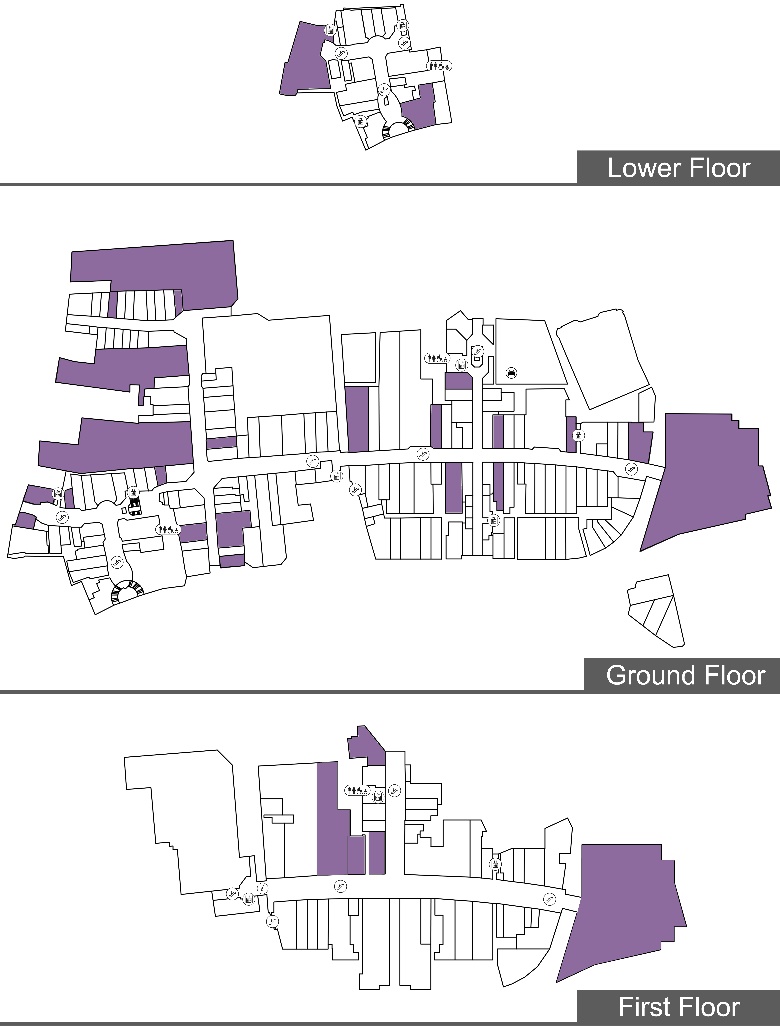


Supplementary Figure 10: The map used to locate recovery spaces with the existing facilities and landmark shops (brand names anonymised for confidentiality)

1. **What is the type of your recovery space?**

- Quiet room
- Sensory room
- Quiet corner
- Outdoor space
- Bench
- Other: …………

Participants who chose a closed space were given a cube model to resemble an empty room. The model was constructed from foam boards and glued on only six edges, allowing it to be easily detached for manipulation (see Supplementary Figure 11). The dimensions of the model were inspired by BS 8300-2 (The British Standards Institution, 2018). It specifies the minimum size of the quiet room as 2.1 m x 2.3 m. The size chosen for the room was 4 m x 4 m, forming a square for easier manipulation in the subsequent design activities. The selected architectural scale was 1:20, which was sufficiently clear for the participants to create detailed designs for their space.


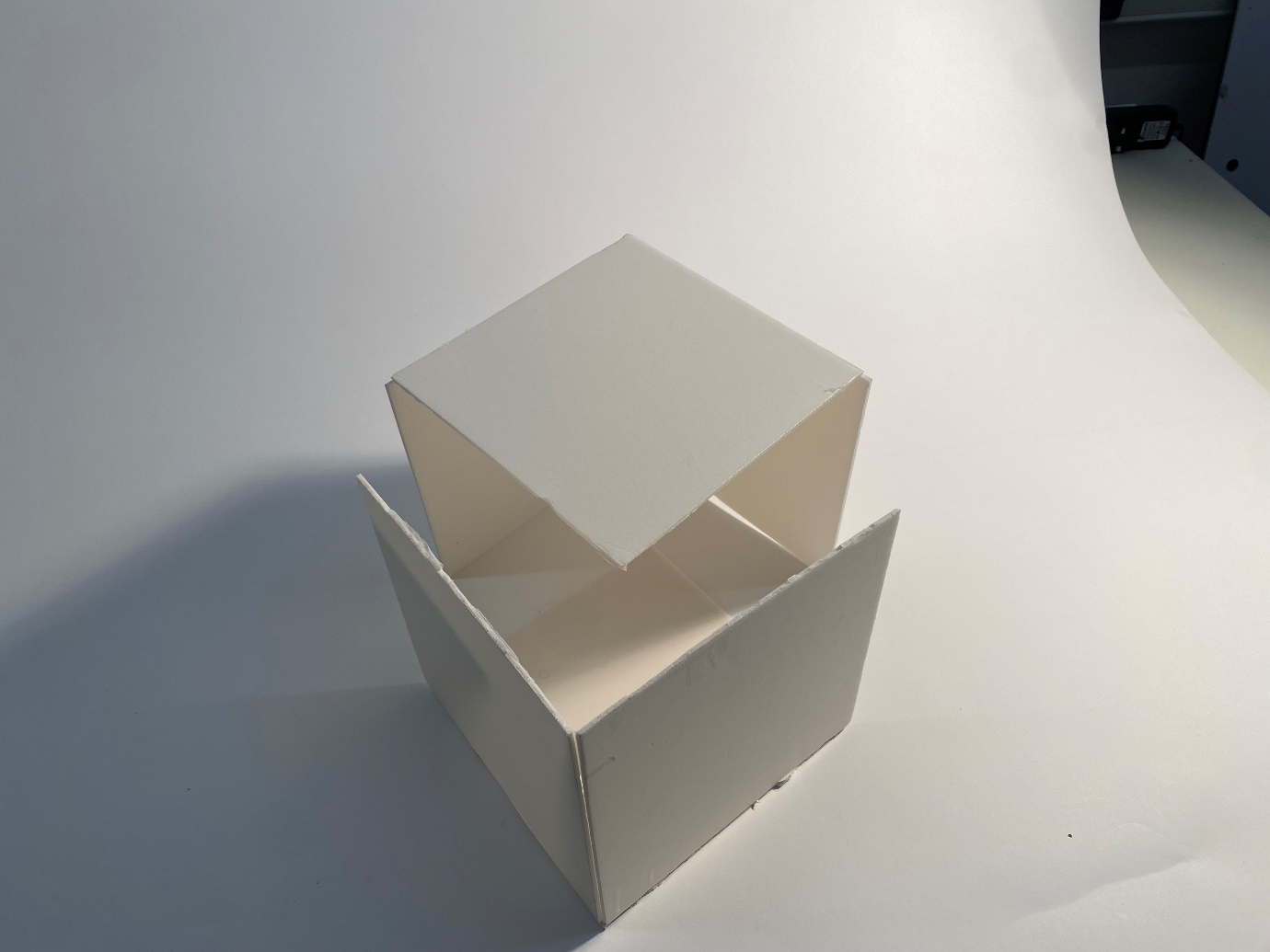


Supplementary Figure 11: Recovery space base model

1. **What are the colours of your space?**

Participants were provided with coloured papers resembling paint to select their wall colours (see Supplementary Figure 12).


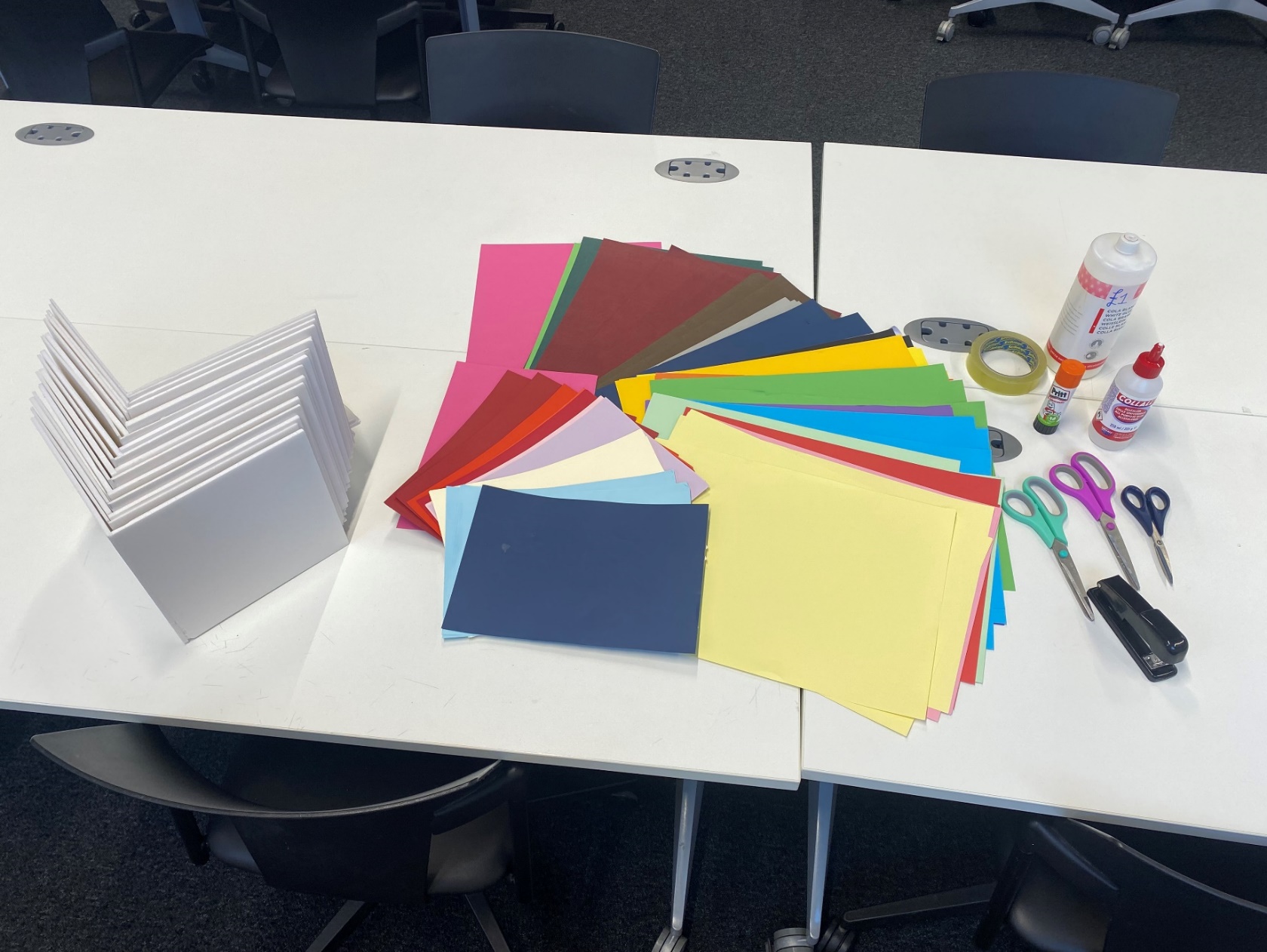


Supplementary Figure 12: Wall colour selection

1. **How does the floor look like? Is it a hard floor or a soft floor?**

Participants were provided with a selection of printed hard floors and soft floors in soft foam and felt (see Supplementary Figure 13).


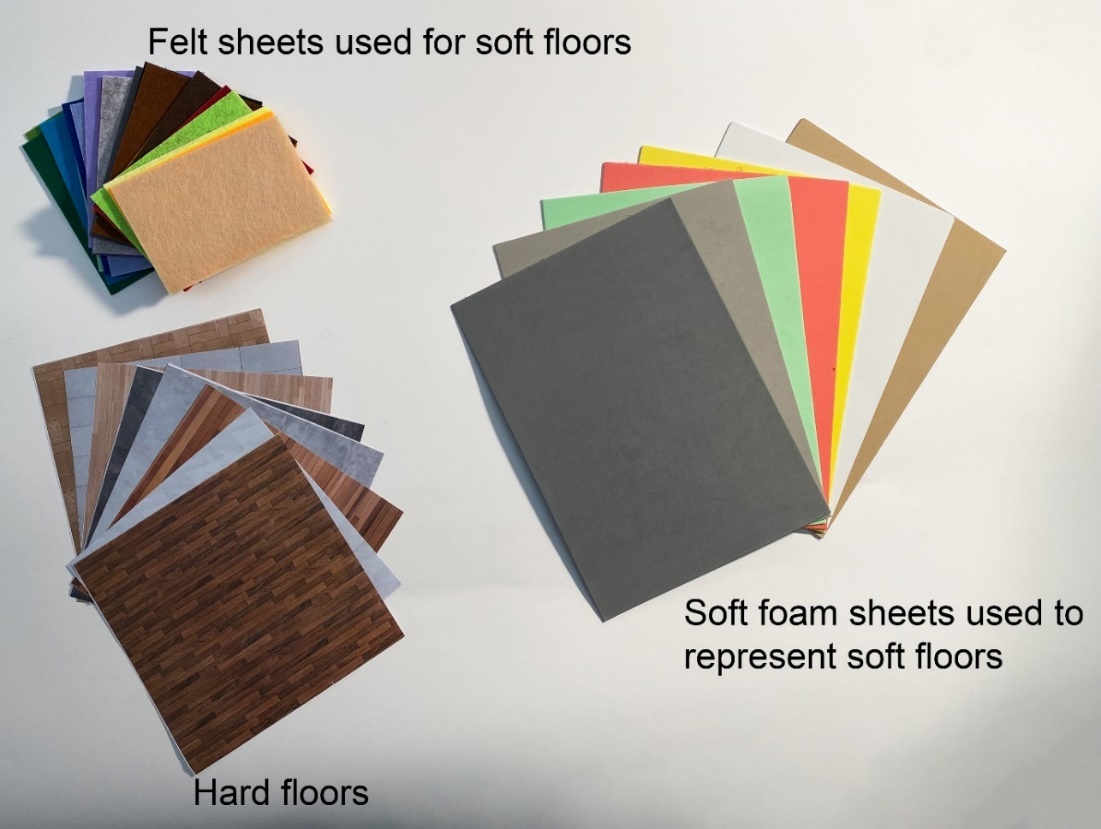


Supplementary Figure 13: Floors selection

1. **Does it have doors, windows or skylights?**

Participants were provided with a printed selection of different doors, windows, skylights, and occupancy indicators for those who wanted to design a single-occupancy room (see Supplementary Figure 14).


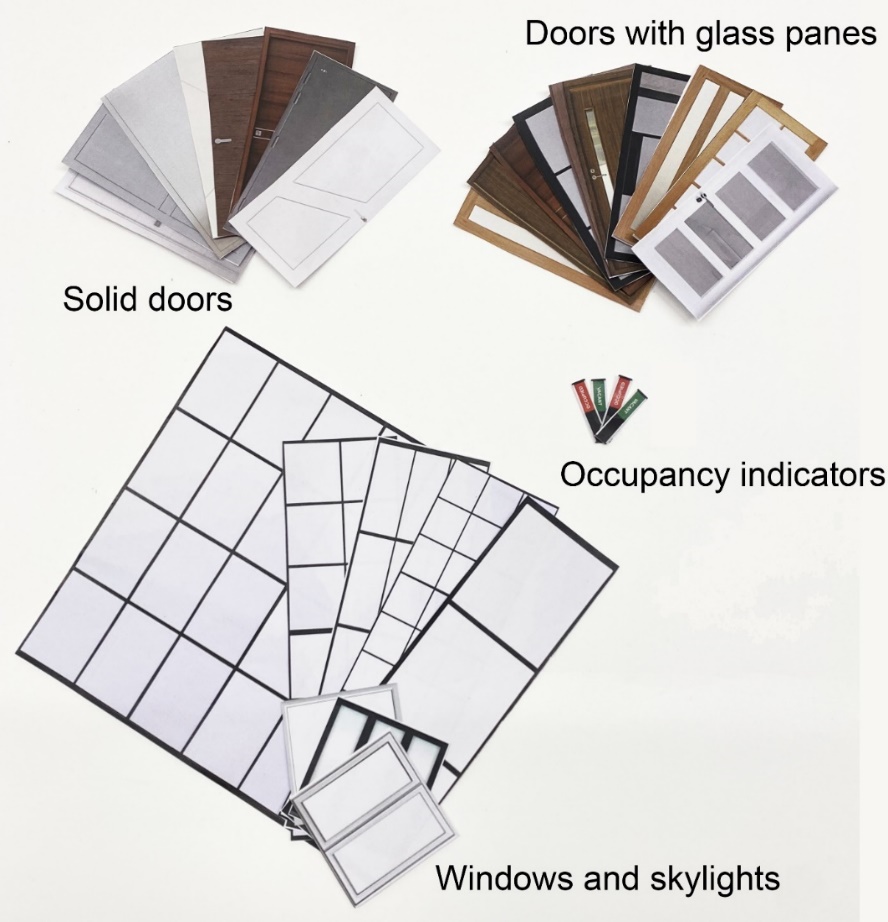


Supplementary Figure 14: Openings selection

1. **What is the furniture like in your room?**

Participants were provided with a 3D-printed selection of different furniture (see Supplementary Figure 15). The furniture selection was inspired by the guidance in PAS 6463:2022 Design for the Mind-Guide (The British Standards Institution, 2022) on quiet spaces furnishing:

- Provide comfort.
- Easily repositioned.
- A variety of movable seating to meet a range of user needs, including informal and lower options, such as beanbags and floor cushions.


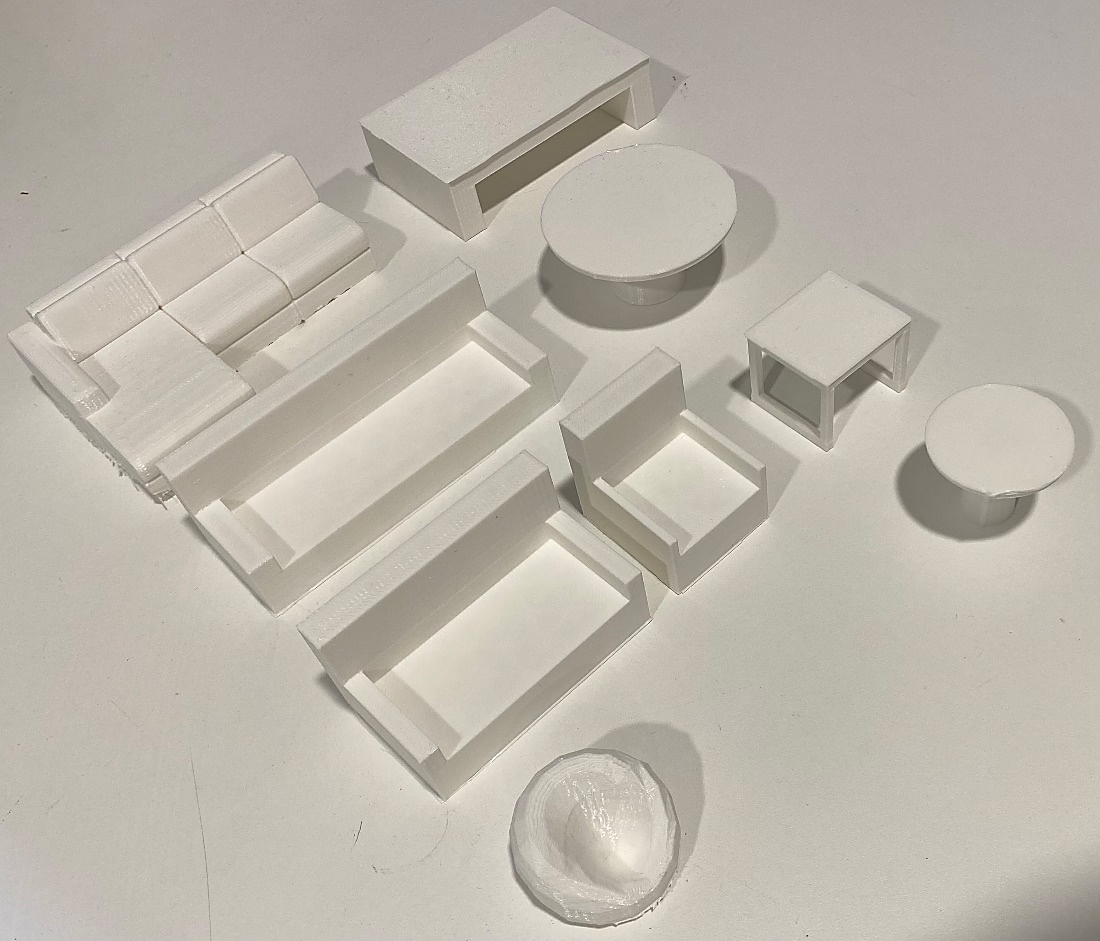


Supplementary Figure 15: Furniture options

1. **Now, describe your design**

Participants were asked to interpret their design and justify their design decisions verbally.

##### Locating facilities

The second activity was to identify areas where additional facilities are needed around the centre as a group. The participants were provided with six A1 maps with landmark shops, informed by the walking interviews (see Supplementary Figure 16). Each map focused on a single facility; the maps were:

- Restaurants and coffee shops
- Electronic maps
- Information desk
- Seats
- Toilets
- Vertical connection nodes
- Other (participants can add any facility they need)


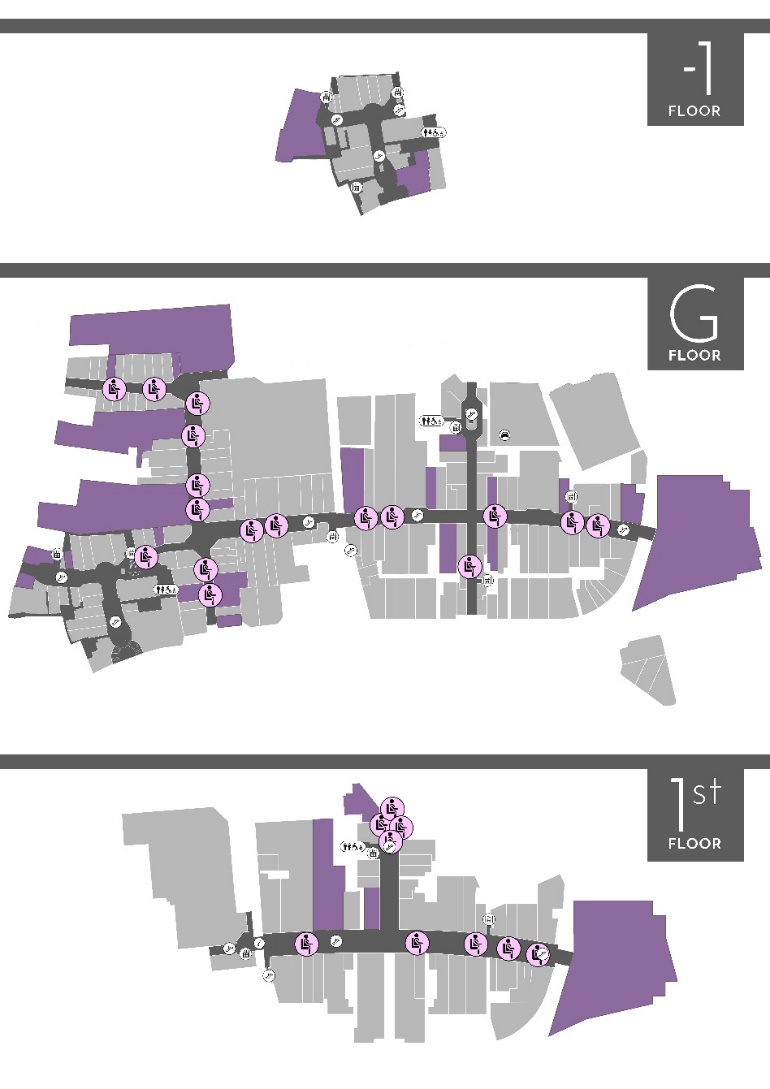


Supplementary Figure 16: An example of the centre maps provided to participants with existing seats, signposted by facilities and landmark shops, which the participants used to locate extra seats where needed (redacted for anonymity)

These categories were chosen based on the results of walking interviews and the focus groups. The participants were also given stickers so they could place them where they needed more facilities. The facilities key on the maps and stickers had the same shape, size, and colour, making it easier for participants to interact with the activity.

##### Designing signs

The last activity was designing signs individually. Like designing recovery spaces activity, the design process was divided into stages with different prompt questions. These questions were also added to the workshop presentation, aided with photos as additional prompts.

1. **Which type of sign are you designing?**

Participants chose what type of sign they wanted to design (see Supplementary Figure 17).


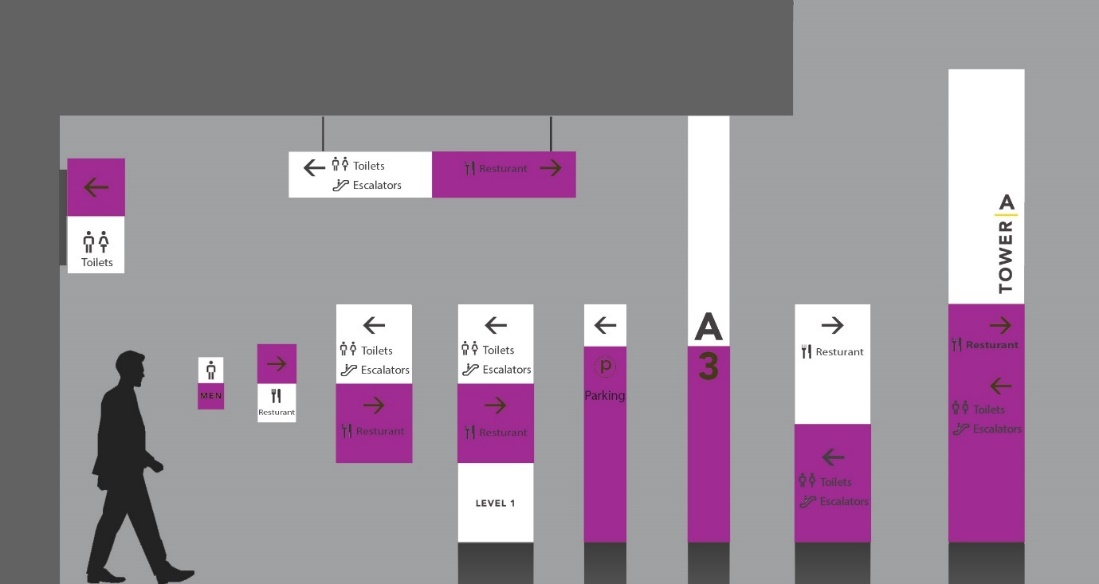


Supplementary Figure 17: Signs types for the participants to choose from

1. **What is the colour of your sign? Is it one colour?**

Participants were provided with coloured papers and pens to select the colour of the signs and text (see Supplementary Figure 18).


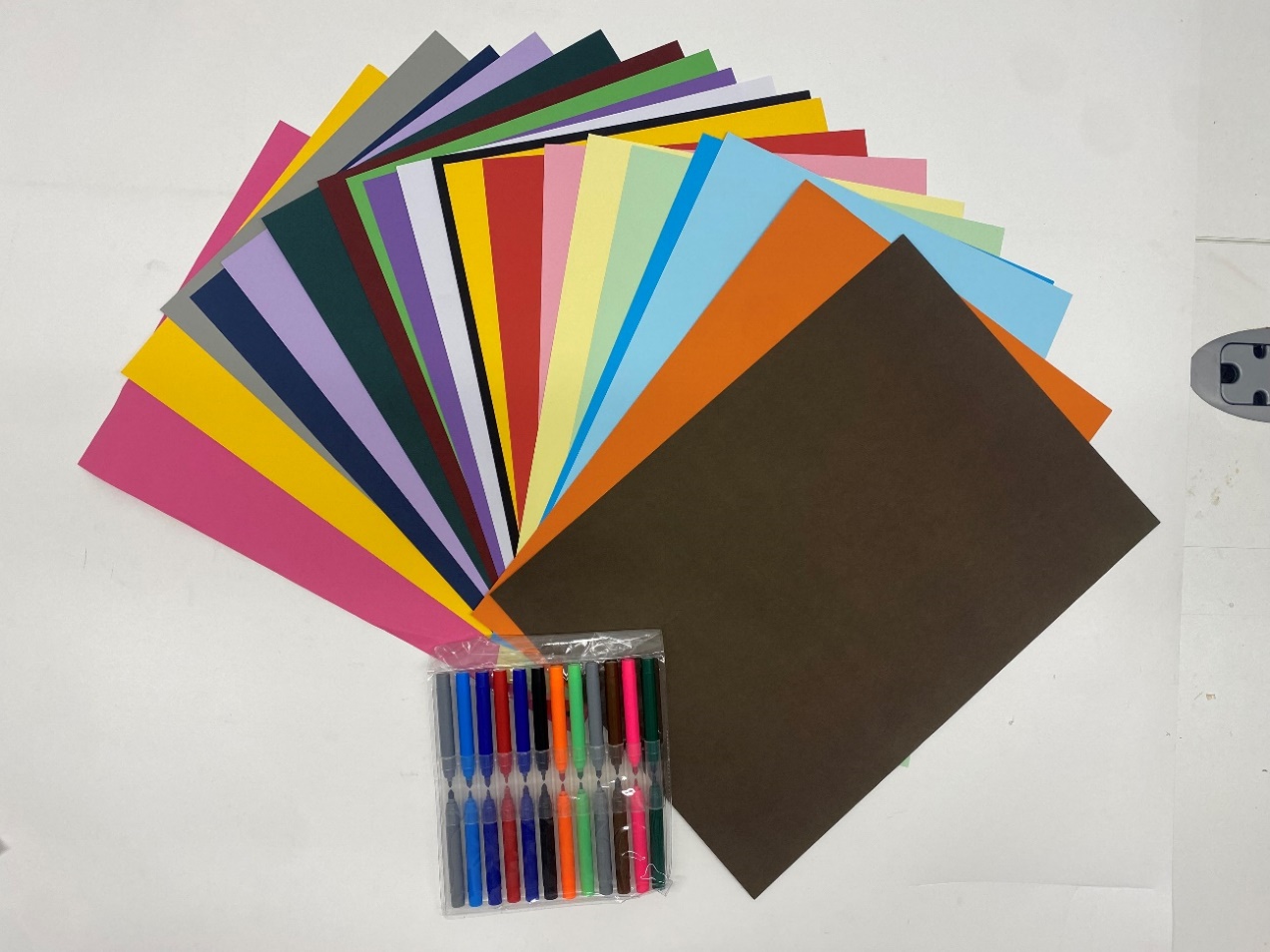


Supplementary Figure 18: Coloured papers and pens for sign designing sign

1. **Don’t forget the Welsh translation**

Participants were provided with the Welsh translation for common signs and asked whether they wanted different colours for Welsh and English or just one colour (see Supplementary Figure 19).


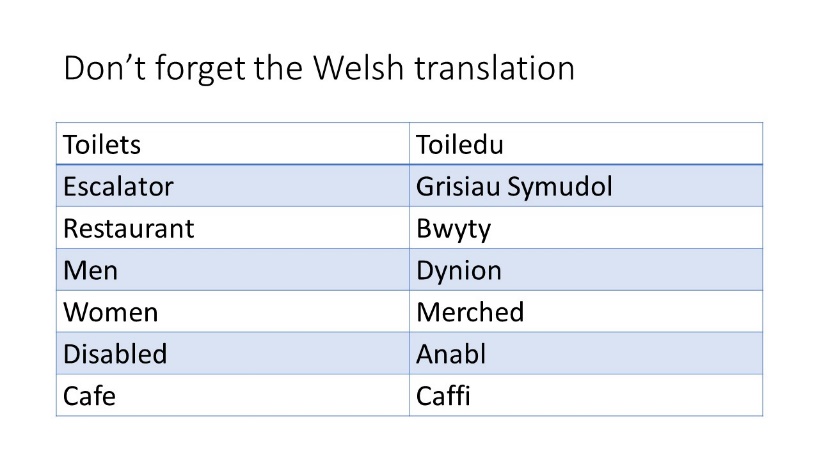


Supplementary Figure 19: Welsh translation for common signs

## References

Gary, B., Amanda, C., Nikoletta, G., & Irene, T.-W. (2012). Doing it together (DM Special Issue) [Article]. *British Journal of Learning Disabilities*, *40*(2), 134–142. https://doi.org/10.1111/j.1468-3156.2012.00744.x

The British Standards Institution. (2018). *BS 8300-2:2018 Design of an accessible and inclusive built environment*.

The British Standards Institution. (2022). *Design for the mind – Neurodiversity and the built environment – Guide*.
